# Conjugation of Antibiotics to Peptidomimetics Enhances Antimicrobial Spectrum of Activity

**DOI:** 10.3390/antibiotics15050484

**Published:** 2026-05-11

**Authors:** Joshua Fleming, Nathan James Carey, Yao Cheng, Hao Luo, Tsz Tin Yu, Mark D. P. Willcox, David StC Black, Edgar H. H. Wong, Naresh Kumar

**Affiliations:** 1 School of Chemistry, The University of New South Wales (UNSW), Sydney, NSW 2052, Australia; josh.fleming@unsw.edu.au (J.F.); nathan.carey@unsw.edu.au (N.J.C.); yao.cheng@unsw.edu.au (Y.C.); d.black@unsw.edu.au (D.S.B.); 2 School of Chemical Engineering, The University of New South Wales (UNSW), Sydney, NSW 2052, Australia; hao.luo4@unsw.edu.au (H.L.); edgar.wong@unsw.edu.au (E.H.H.W.); 3 School of Optometry and Vision Science, The University of New South Wales (UNSW), Sydney, NSW 2052, Australia; m.willcox@unsw.edu.au

**Keywords:** AMP mimic, antimicrobial conjugate, peptidomimetic, fluoroquinolone, cephalosporin, SMAMP, antimicrobial resistance

## Abstract

**Background/Objectives:** Antimicrobial conjugates have attracted considerable interest in addressing the threat of antimicrobial resistance by minimising the likelihood of resistance onset. Antimicrobial peptide mimic–antibiotic conjugates offer a unique strategy to revitalise current clinical agents through increased membrane permeabilisation, prolonging the longevity of traditional antibiotics while broadening the spectrum of activity of the AMP mimic. **Methods:** This study explored non-cleavable, enzyme-cleavable, and pH-cleavable linked conjugates between an anthranilamide-based peptide mimic and current clinically available antibiotics to assess the viability of conjugation in enhancing antimicrobial activity as measured through MIC assays. Cleavage studies were conducted to assess the stimulus susceptibility of relevant compounds. **Results:** Four amide-linked non-cleavable conjugates were synthesised. Of these, a primary amide-linked conjugate between ciprofloxacin and the peptidomimetic had the most significant activity with an MIC of 15.6 µM towards Gram-positive *Staphylococcus aureus* and Gram-negative *Pseudomonas aeruginosa*, and an MIC of 7.8 µM towards Gram-negative *Escherichia coli*. A hydrazone-based pH-sensitive linker system was synthesised and had an MIC of 15.6 µM towards Gram-negative *E. coli*. Finally, an enzyme-cleavable cephalosporin conjugate system was investigated, which offered a unique method for the specific treatment of resistant bacterial strains. Cleavage studies of this conjugate suggested rapid degradation of the β-lactam ring and release of the subunit. **Conclusions:** This work presents conjugate systems between peptide mimics and antibiotics as a new, promising strategy to broaden the antimicrobial spectrum of novel antimicrobial agents.

## 1. Introduction

Antimicrobial resistance (AMR) amongst pathogenic bacteria poses a significant global health threat [1]. Mechanisms of AMR most commonly encountered include structural modification of the antibiotic’s targeted site, upregulation of antibiotic degradation enzymes, increased expression of efflux pumps, and modulation of frequency or selectivity of porin channels in the outer membrane of Gram-negative bacteria [1,2,3]. Traditional antibiotics often have a single target or mechanism of action (MOA), and so are more susceptible to resistance across a class, necessitating the development of novel antimicrobial therapeutics with new, robust mechanisms of action [4].

Antimicrobial peptides (AMPs) are involved in the first line of the human immune defence [5]. Due to their primarily membrane-active MOA of permeabilising the bacterial cell membrane, which is an evolutionarily conserved region of the cell, they are believed to be less susceptible to resistance; hence, AMPs are an effective avenue for combating AMR [6,7]. However, AMPs often suffer from poor bioavailability, proteolytic degradation, and are expensive to synthesise or isolate, which has prevented them from becoming a widespread treatment strategy [8,9,10].

**Figure 1:**
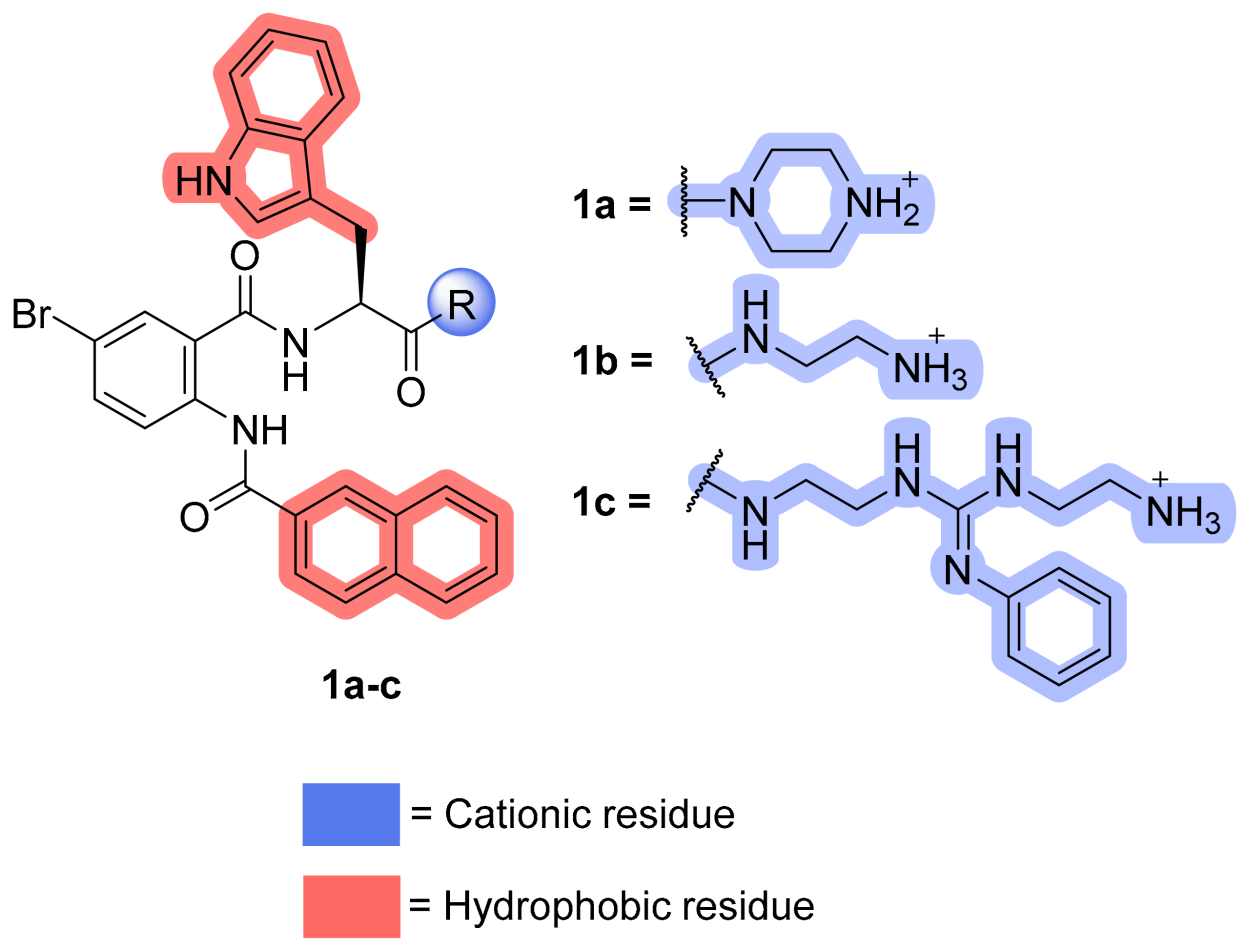
Anthranilamide SMAMP scaffold with the amphipathic structure highlighted: hydrophobic tryptophan and naphthalene residues (red) and hydrophilic cationic residue (blue).

Synthetic mimics of antimicrobial peptides (SMAMPs) are small molecules denoted by their amphipathic structure, which retain the MOA of AMPs without the associated limitations, making them promising candidates in the development of novel antimicrobial agents [11,12]. The amphiphilicity of SMAMPs is essential to their antimicrobial activity as the cationic residue of the compound interacts with anionic phospholipids of bacterial cell membranes preferentially to zwitterionic mammalian cells. Upon this interaction, the hydrophobic portion can insert and permeate the bacterial cell membrane, forming pores to disrupt ion gradients and cell integrity, ultimately leading to cell death [13]. Our group has seen success in the development of antimicrobial compounds against Gram-positive bacteria using an anthranilamide peptidomimetic scaffold, bearing tryptophan and naphthyl hydrophobic residues and a hydrophilic cationic ammonium group (Figure 1) [14,15].

Despite the potency of the anthranilamide derivatives against Gram-positive bacteria such as *S. aureus*, they have had more limited efficacy against Gram-negative bacteria such as *E. coli* and *P. aeruginosa,* which pose a greater threat due to increased resistance afforded by the additional outer lipid membrane [16]. The limited efficacy toward Gram-negative strains necessitates the investigation of strategies that can broaden the spectrum of activity of the peptidomimetic, with conjugation of two antimicrobial subunits serving as a promising potential solution.

Multimodal warheads made through the conjugation of antibiotics have been shown to provide efficacy greater than that of co-administration of multiple antimicrobial agents. This can be attributed to conjugation changing the fundamental pharmacokinetic properties of the compound [17]. Drug conjugates have already proven to be a viable strategy in other areas of medicine, including the conjugation of drugs to other drugs, antibodies, and adjuvants [18,19]. Recent work has explored the conjugation of antibiotics to efflux pump inhibitors [20], nitric oxide donors [21], siderophores [22,23] and traditional AMPs [17,24,25]. Adjuvant conjugate systems often use cleavable linkers that are labile to pH or enzymatic degradation characteristic of bacterial cells, to allow for full antimicrobial functionalisation upon internalisation [22]. Alternatively, non-cleavable linkers have also been used in the formation of dual-action conjugates to minimise the risk of premature release [26]. Despite conjugation being an effective strategy to overcome the shortcomings of antibiotics, there has been limited investigation into SMAMP—antibiotic conjugates.

A previously synthesised anthranilamide peptide mimic, RK-758 (**1c**), has been observed to act synergistically with fluoroquinolone antibiotic ciprofloxacin upon coadministration, making this peptide mimic scaffold an ideal candidate for assessing the feasibility of conjugation between SMAMPs and antibiotics [7]. Conjugation is a superior strategy to co-administration as it addresses the translation problem between in vitro and in vivo efficacies due to differing pharmacokinetics in vivo, allowing for the drug development process to be streamlined [27]. Through drug profile unification, the synergistic activity of the antimicrobial agents is maximised, ensuring that selective pressures are placed on several targets simultaneously to reduce the risk of resistance development [25].

**Figure 2:**
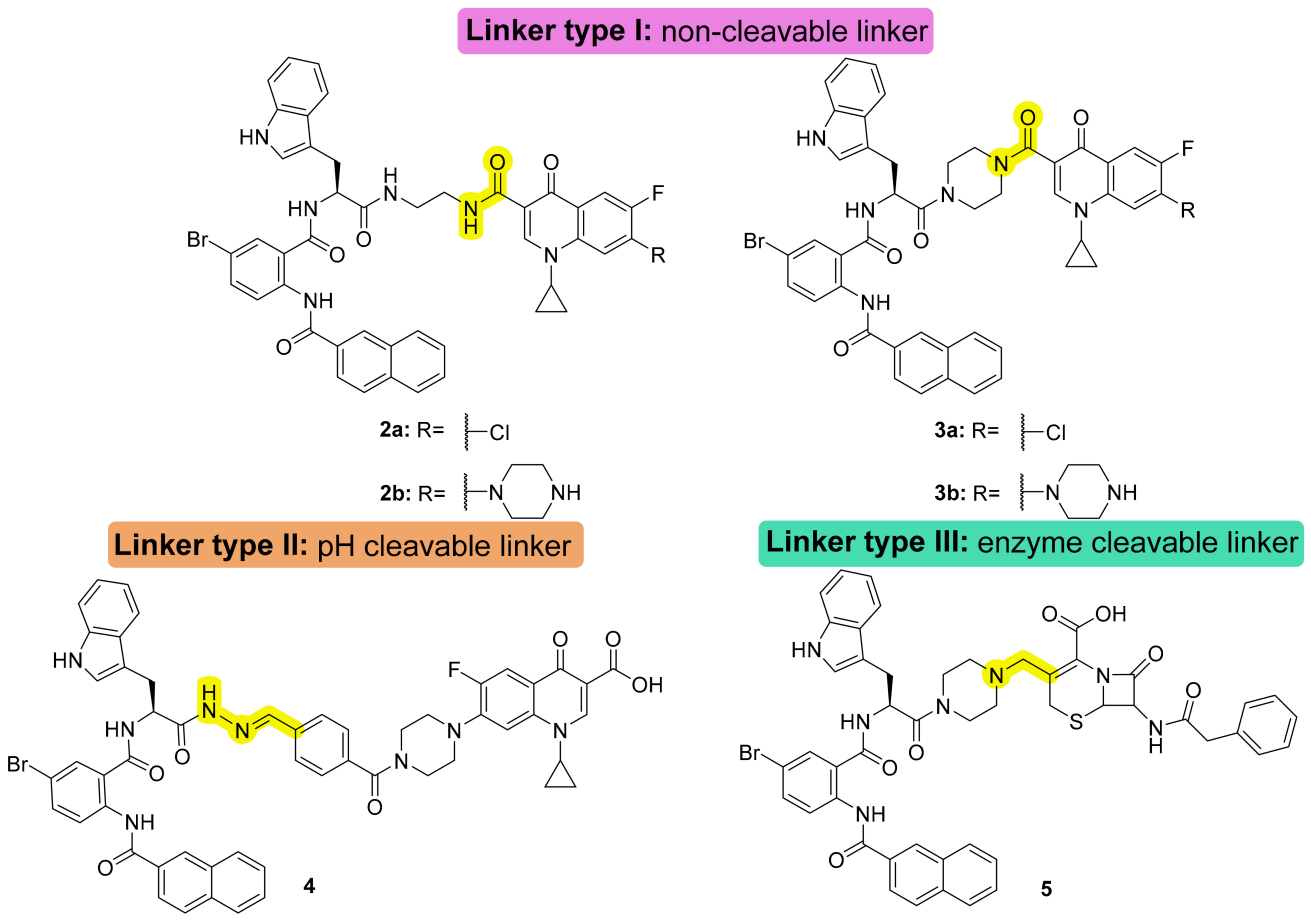
Structures of non-cleavable (**2a**–**2b**, **3a**–**3b**), pH-cleavable (**4**), and enzyme-cleavable (**5**) conjugates, with the linker highlighted in yellow.

This work aimed to assess the viability of conjugation in enhancing the antimicrobial activity of SMAMPs and antibiotics. A simplified anthranilamide scaffold was tested, rather than the lead RK-758 (**1c**), to verify if conjugation was an effective strategy prior to guanidinylation. Similarly, increased membrane permeabilisation should revitalise the antibiotic by minimising the effect of key resistance mechanisms like efflux pump expression. In this study, we explored an amide-based non-cleavable linker system and a hydrazone-based acid-labile linker system between fluoroquinolone antibiotics and anthranilamide peptide mimics. We further investigated an enzyme-cleavable system between an anthranilamide peptide mimic and a cephalosporin antibiotic, owing to the enzymatic susceptibility of the β-lactam ring as a common resistance mechanism towards β-lactam antibiotics (Figure 2) [28]. The antibacterial activity of each of the conjugates was assessed using MIC assays, and cleavage studies were conducted on relevant conjugate systems to assess linker stability.

## 2. Results and Discussion

### 2.1. Synthesis of Conjugates

#### 2.1.1. Synthesis of Anthranilamide Peptide Mimic

**Scheme 1:**
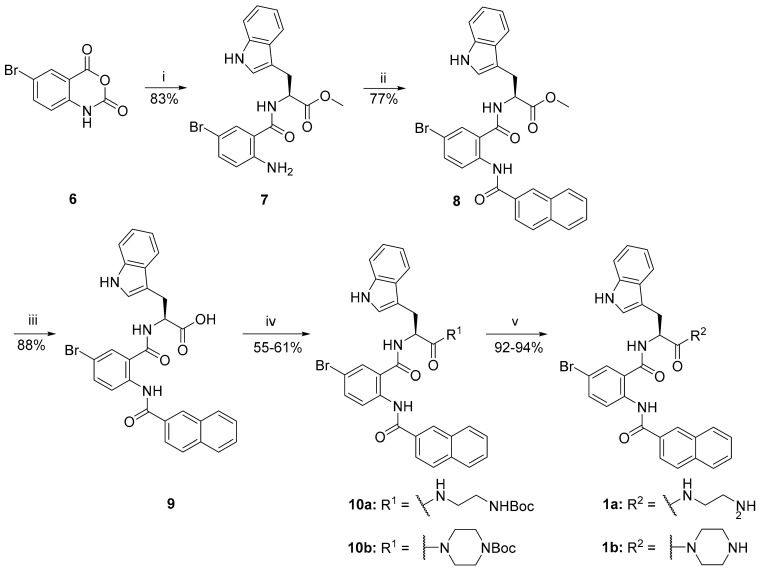
General synthetic scheme for peptide mimic synthesis **1a**–**1b**. Reagents and conditions: (i) 5-bromoisatoic anhydride (1 equiv.), methyl-L-tryptophanate (1.0 equiv.), CH_3_CN, reflux, 16 h; (ii) 2-naphthoyl chloride (1.0 equiv.), Et_3_N (3.0 equiv.), CH_2_Cl_2_, r.t., 4 h; (iii) 1 N NaOH(aq) (2.0 equiv.), THF, MeOH, r.t., 8 h; (iv) Boc-protected amine (1 equiv.), EDC (1.2 equiv.), HOBt (1.2 equiv.), DIPEA (2.5 equiv.), DMF, r.t., 16 h; (v) 4M HCl/dioxane, CH_2_Cl_2_, r.t., 4 h.

Synthesis of the peptide mimics **1a–1b** was previously reported by Kuppusamy and coworkers, through the ring-opening of 5-bromoisatoic anhydride **6** with tryptophan methyl ester in 83% yield, followed by amidation at the anthranilamide amine **7** with 2-naphthoyl chloride to give compound **8** in 77% yield. Subsequently, a cationic residue was introduced through amidation at the hydrolysed tryptophan C-terminus of compound **9**, with a Boc-protected diamine in 48–54% yield over two steps. Exposure of compound **10** to the acid cleaved the Boc group to provide the desired peptide mimic **1a**–**1b** in 92–94% yield (Scheme 1) [14].

#### 2.1.2. Synthesis of Non-Cleavable Linked Conjugates

**Scheme 2:**
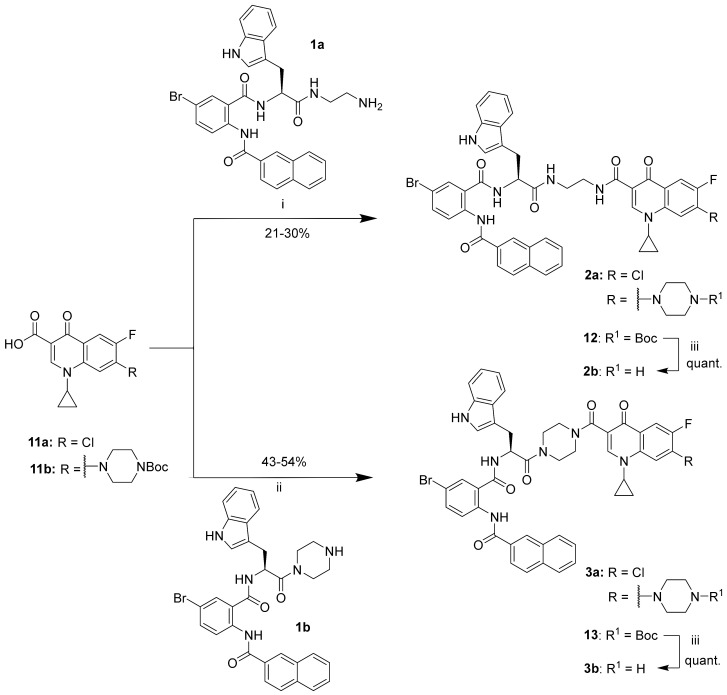
General synthetic scheme for amide conjugates **2a**–**2b** and **3a**–**3b**. Reagents and conditions: (i) **1a** (1 equiv.), EDC (1.5 equiv.), HOBt (1.5 equiv.), DIPEA (2.0 equiv.), DMF, r.t., 18h. (ii) **1b** (1.0 equiv.), HATU (1.2 equiv.), DIPEA (2.0 equiv.), DMF, r.t., 18h. (iii) 4 M HCl/dioxane (2 equiv.), CH_2_Cl_2_, r.t., 6h.

An amide linker system was selected for the non-cleavable class, owing to its high degree of stability under physiological conditions. They have been used to conjugate AMPs and antibiotics, further supporting their capability as linkers of antimicrobial agents [26,29]. To assess the effect of molecular size, the fluoroquinolone substrate was varied between a piperazine and a chloro-substituent at C7. Further, both primary and secondary amide conjugation strategies were considered to modulate hydrophobicity and flexibility at the linkage site. This consideration is essential, as the non-cleavable class will inherently have a region masked in conjugation, considerably affecting the conjugates’ efficacies. Amidation proceeded under EDC/HOBt or HATU-mediated coupling between the carboxylic acid group of the fluoroquinolone and the terminal amino group of the peptidomimetic **1a**–**1b** to yield conjugates **2a**, **3a,** and protected conjugates **12**–**13** in 21–54% yield. Following this, the piperazine-bearing fluoroquinolone conjugates (**12**–**13**) were Boc-deprotected under acidic conditions to yield the free amine **2b** and **3b** quantitatively (Scheme 2).

#### 2.1.3. Synthesis of Acid-Cleavable Linker

Hydrazone linkers have been reported as acid-labile systems. They are highly stable under physiological conditions, but are rapidly hydrolysed at the site of infection due to the acidic environment proximal to infection sites [19,30,31]. Generation of a hydrazone linker required modification of both antimicrobial agents to bear either a formyl or hydrazide group. Substitution of the ethyl-amine chain of peptidomimetic **1a** with a hydrazide group was identified as a convenient method to introduce the desired moiety, as the predicted cleavage product would retain the cationic residue functionality, which is essential for the AMP mimic’s activity.

**Scheme 3:**
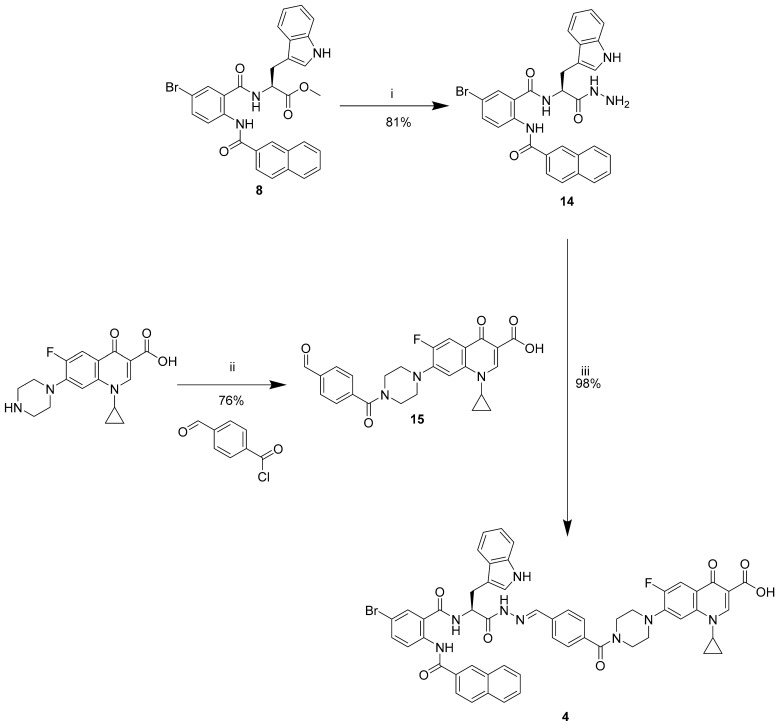
Synthesis of modified antimicrobial agents **14** and **15** and conjugate **4**. Reagents and conditions: (i) hydrazine monohydrate (3.0 equiv.), MeOH, THF, reflux, 18 h. (ii) 4-Formylbenzoyl chloride (1.0 equiv.), Et_3_N (4.0 equiv.), anhydrous CH_2_Cl_2_, r.t., 18 h. (iii) **15**, HCl (cat.), MeOH, reflux, 0.5 h.

To form the peptidomimetic fragment of the conjugate, methyl ester intermediate **8** was refluxed with hydrazine monohydrate, forming **14** in 81% yield. The fluoroquinolone fragment was synthesised by amidation of ciprofloxacin with 4-formylbenzoyl chloride to generate the corresponding **15** in 76% yield. Hydrazide **14** and aldehyde **15** were refluxed in acidic conditions to afford the hydrazone conjugate **4** quantitatively (Scheme 3).

#### 2.1.4. Synthesis of Enzyme Cleavable Linker

**Scheme 4:**
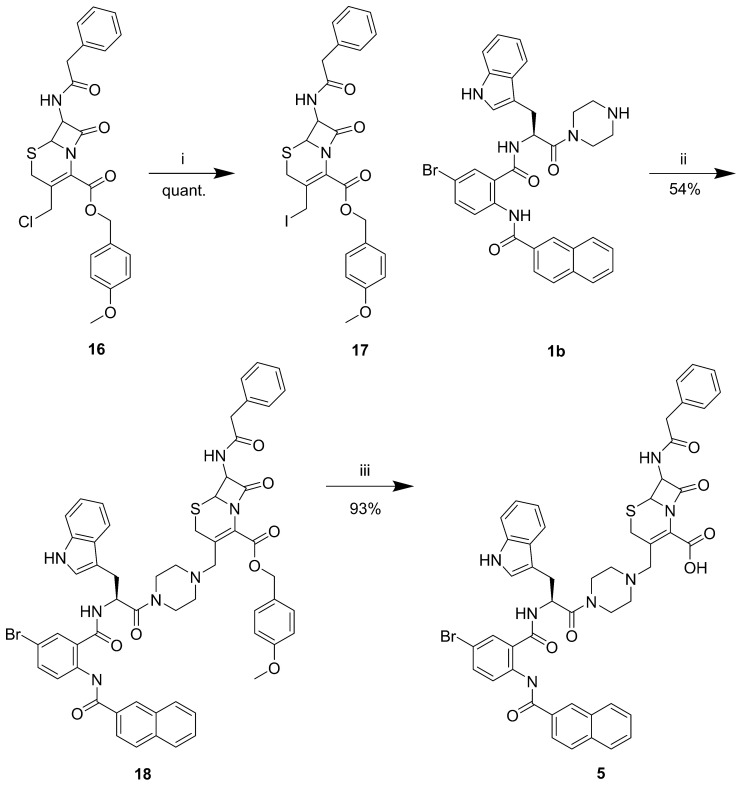
Synthesis of antimicrobial conjugate **5**. Reagents and conditions: (i) NaI (1.0 equiv.), acetone, r.t., 6 h. (ii) Na_2_CO_3_, DMF, 0 °C, r.t., 2 h. (iii) Trifluoroacetic acid (TFA), thioanisole, 0 °C, r.t., 2 h.

β-lactam antibiotics such as cephalosporins are particularly vulnerable to enzymatic degradation. The susceptibility of cephalosporins to β-lactamase enzymes makes them a promising candidate in the generation of conjugated dual-action antimicrobials by utilising the degradation pathway to open the four-membered ring, releasing an amine-bearing antimicrobial subunit [28,32]. The conjugate was synthesised through halogen metathesis between a PMB-protected chloro-cephalosporin moiety (**16**) and sodium iodide, followed by *N-*alkylation with the piperazine peptide mimic (**1b**). After subsequent deprotection of the PMB group under acidic conditions, the desired conjugate **5** was isolated in 49% yield over two steps (Scheme 4).

### 2.2. Cleavage Studies

#### 2.2.1. Hydrazone Acid Cleavage Studies

Hydrazone conjugate **4** was anticipated to undergo cleavage under acidic conditions to release the hydrazide-modified peptide mimic **14** and formylated ciprofloxacin **15**. To verify the hydrolytic susceptibility of the hydrazone linker, a series of ^1^H NMR experiments was conducted, with varying equivalents of acid. Spectra were taken at regular intervals and monitored through the appearance of the formyl proton of the released formylated ciprofloxacin **16** to assess the instability of the hydrazone bond, as summarised in Table 1.

Conjugate **4** was subjected to a series of ^1^H NMR experiments in an acidic environment with concentrated HCl, at 10, 30, and 50 equivalents added to the sample for ^1^H NMR analysis. Spectra were taken in DMSO-*d*_6_ due to the limited solubility of **4**; however, with the addition of conc. HCl, there was still an excess of water to facilitate hydrolysis. Cleavage studies indicate that with increasing acid equivalences, the hydrolysis proceeds at a significantly faster rate. For instance, at 10 equivalents of HCl, only approximately 9% of the conjugate is released after 30 min. In contrast, at 50 equivalents of HCl, 29% of the conjugate is released after 30 min. This trend is more obvious at longer incubation time, with the percentage of release of conjugate in 10 equivalents of HCl only reaching 23% after 72 h, less than that of 28% after 30 min in 30 equivalents of acid.

Ideally, the release of the conjugated species should occur slowly in moderately acidic and neutral environments. The conjugate possesses this stability as there is only mild release of the conjugated species after 72 h, even at 10 equivalents of acid (Table 1). However, at increasing molar equivalence of acid, the conjugate remains relatively stable, with over half remaining conjugated, indicating a stability about the hydrazone bond. To make the nucleophilic attack of water more effective, an alkyl spacer may be introduced between the peptide mimic moiety and the hydrazone to remedy steric bulk about the hydrazone bond. Further, through the addition of an alkyl spacer, the linker system would no longer be an acyl hydrazone, which is characterised by a greater degree of stability due to the increased electronegativity of C_1_, reducing electrophilicity [33].

Despite the complete release of the conjugate not being evident, conjugate **4** still had a significant hydrolysis dependence on the surrounding acidic environment, presenting itself as a promising linker system to specifically release antimicrobial agents within a bacterial cell lysosome.

#### 2.2.2. β-Lactam Enzyme Cleavage Studies

**Figure 3:**
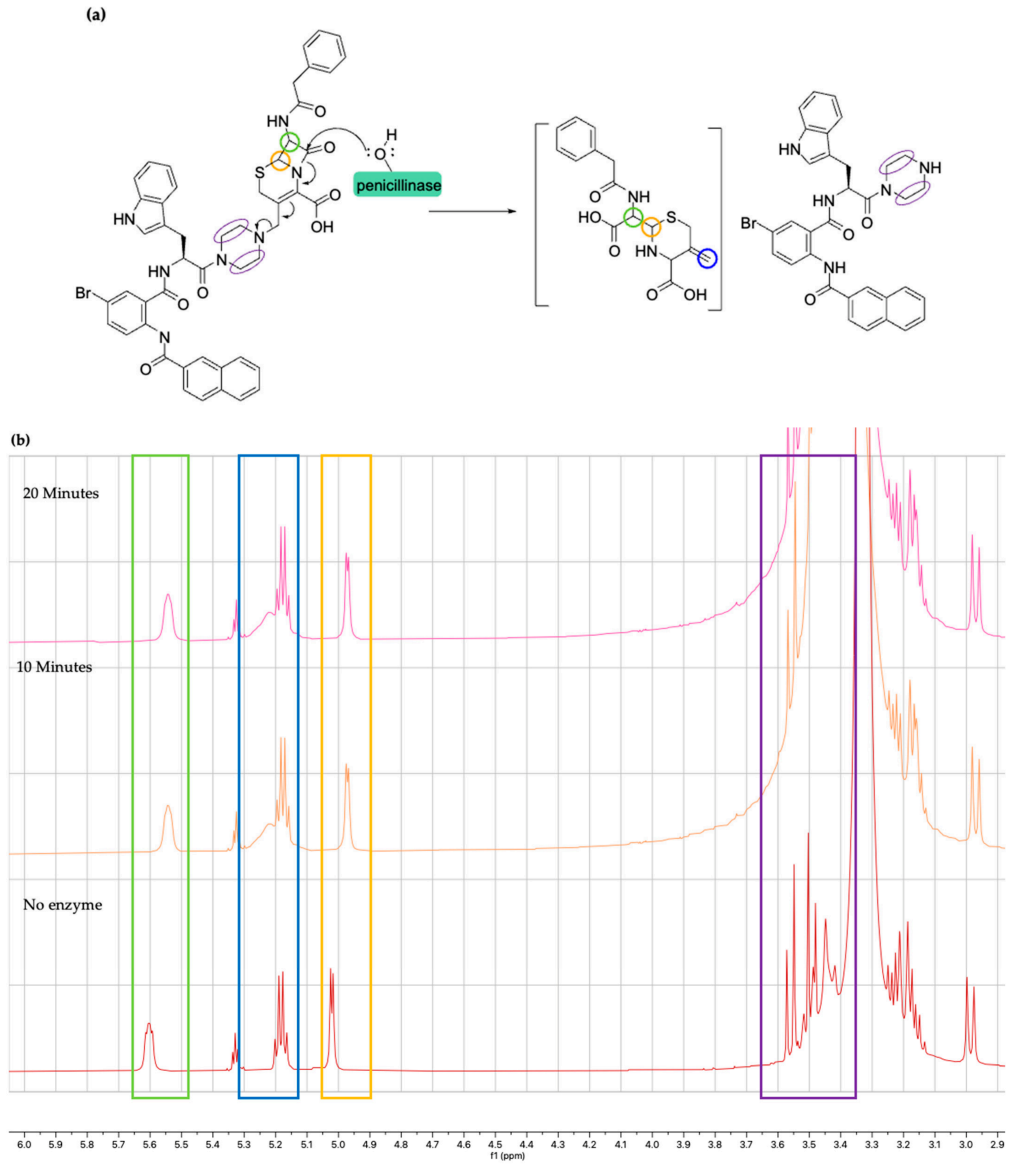
(**a**) Mechanism of ring opening of cephalosporin four-membered ring after enzyme addition, with corresponding changing proton environments highlighted. (**b**) ^1^H NMR (600 MHz, DMSO-*d*_6_) spectrum of conjugate **5** prior to addition of penicillinase (red), 10 min after enzyme addition (Orange), and 20 min after enzyme addition (Pink), with regions of product cleavage highlighted; no significant change to spectra is evident after this point.

The release mechanism for the cephalosporin conjugate **5** has been previously reported in macromolecular dendron conjugates, which exhibited rapid degradation upon exposure to penicillinase (Figure 3a) [32]. To investigate this behaviour, ^1^H NMR (DMSO-*d*_6_) spectra of a solution of **5** were recorded at various time points ranging from 10 min to 3 days following the addition of penicillinase enzyme. NMR results suggested complete cleavage of the conjugate within the initial 10 min period after the addition of the enzyme, with no significant difference in spectra after this point (Figure 3b). This is consistent with previously reported enzymatic degradation of these systems, which suggested rapid and complete cleavage of conjugates within similar timeframes [32,34].

The first spectrum, taken after 10 min, showed an upfield shift in the β-lactam ring at 5.60 ppm (green) and 5.02 ppm (yellow) corresponding to the CH-NH and CH-S protons, respectively. Shifts in ppm are characteristic of the four-membered ring opening, supporting the susceptibility of the conjugate species towards β-lactamase enzymes. The appearance of a new broad singlet at 5.11–5.28 ppm (blue) is likely due to the hydrolysis of the terminal olefin intermediate after nucleophilic attack. Finally, the multiplet at 3.50 ppm (purple) corresponds to the piperazine protons of the peptidomimetic, which also undergo an upfield shift consistent with the differing proton environments of the bound and free amine compounds. Each of these changes supports the degradation of the β-lactam ring and the release of the peptidomimetic within the initial 10 min time frame.

To confirm that the new signals observed in the ^1^H NMR spectra were due to the enzymatic cleavage of the conjugate, the NMR sample was concentrated and subjected to LCMS analysis. The LCMS trace verified almost complete cleavage and recovery of the peptide mimic **1b** (Figure S1). Together, the LCMS and ^1^H NMR data confirmed rapid and near-complete cleavage of the conjugate in the presence of enzymatic stimulus, an outcome highly desirable for the design of enzyme-responsive drug conjugates.

### 2.3. Antimicrobial Activity (MIC)

The antimicrobial activity of the synthesised conjugates was determined by the minimum inhibitory concentration (MIC) assay. For conjugates utilising a labile linker, the MIC of the corresponding cleaved products was also determined to assess the antibacterial activity of the modified released products and infer the significance of cleavage within bacterial media. Determination of MIC for anticipated cleaved products also gave an indication of the synergistic activity of conjugate systems. The MIC values are summarised in Table 2.

#### 2.3.1. Antimicrobial Activity of Non-Cleavable Linked Conjugate

The ciprofloxacin-bearing conjugates **2b** and **3b** had moderate–high activity against Gram-positive *S. aureus* and Gram-negative *E. coli* with MICs of 7.8–15.6 µM. In contrast, fluoroquinolonic acid conjugates **2a** and **3a** had no activity towards either strain at the highest tested concentration of 250 µM. The disparity suggests that the conjugate’s antimicrobial activity is dependent on the piperazine substitution. This could potentially be a result of the cationic amine present facilitating specificity and the initial interaction with the bacterial cell membrane, in a similar fashion to the cationic piperazine of **1b**. Due to the inherent difference in antimicrobial activity between ciprofloxacin and fluroquinolonic acid, conjugates **2a**–**3a** resulted in lower activity. Further, with the cationic group of the peptide mimic being masked in conjugation, and the substitution of the secondary amine with a halogen in fluoroquinolonic acid conjugates **2a**–**3a**, the specificity may be significantly reduced, resulting in a loss of activity.

Interestingly, the primary amide conjugates **2a**–**2b** had moderate–high activity against Gram-negative *P. aeruginosa*, while the secondary amides **3a**–**3b** had no activity. We speculate that the substantial variance may result from the enhanced flexibility of the primary amide ethyl linker, allowing for the fluoroquinolone moiety to function more freely and exert bactericidal activity. Due to the more restrictive nature of the piperazine spacer, the flexibility could be reduced, inhibiting the interaction between the fluoroquinolone and its biological targets. Another potential explanation for the significant increase in activity across this class may be the enzymatic susceptibility of the linker amide. The increased activity could be attributed to cleavage and release of the fluoroquinolone and peptide mimic moieties, allowing them to exert their MOA fully upon internalisation. Due to the non-natural nature of the secondary amide bond and the increased steric bulk about the amide site, conjugates **3a**–**3b** are less susceptible to enzymatic degradation in *P. aeruginosa* medium, resulting in a loss of activity. The high activity of ciprofloxacin conjugates **2b** and **3b** towards Gram-negative bacteria *E. coli*, and primary amide conjugates **2a**–**2b** towards *P. aeruginosa*, highlights the capability of conjugation as a strategy to broaden the spectrum of activity of the antimicrobial peptide mimics, which were previously ineffective in treating Gram-negative bacterial strains.

#### 2.3.2. Antimicrobial Activity of Acid-Cleavable Linked Conjugate

The hydrazone conjugate **4** is anticipated to yield the hydrazide peptide mimic **14** and formyl ciprofloxacin **15** upon cleavage. The antimicrobial activity of the cleaved products in isolation was determined to assess if the conjugate increases antimicrobial activity, and if the antimicrobial activity of the subunits was affected through modification. Formylated ciprofloxacin **15** had MICs of 7.8 µM against *S. aureus* and *E. coli*, retaining its antimicrobial potency. It also retained activity against *P. aeruginosa* with an MIC of 31.3 µM. It was anticipated that with full hydrolysis of the hydrazone bond, the activity of the conjugate should reflect the MIC of the ciprofloxacin moiety upon release. Interestingly, the hydrazide-modified peptide mimic **14** had no activity across the bacterial strains tested. This indicates that the reduction in alkyl chain length of the peptide mimic was detrimental to its antimicrobial activity. The addition of an alkyl chain spacer may allow for the activity to be revitalised whilst also increasing the hydrolytic susceptibility of the conjugate system.

Conjugate **4** had an MIC of 7.8 µM against Gram-positive *S. aureus*, which is consistent with the release of the ciprofloxacin moiety in the cell lysosome, allowing for the fluoroquinolone to exert its antimicrobial activity. Similarly, conjugate **4** had an MIC of 15.6 µM towards Gram-negative *E. coli*, with only a slight reduction compared to formyl ciprofloxacin **15** on its own. The similar antimicrobial profile in both *S. aureus* and *E. coli* indicates that the conjugate can effectively permeate the cell membrane of these strains and release the antimicrobial subunits within the lysosome. In contrast, conjugate **4** had minimal activity towards *P. aeruginosa*. Despite this, the formyl-modified ciprofloxacin subunit **15** in isolation had an MIC of 31.3 µM against this strain. The loss of activity suggests insufficient cleavage of the conjugate species within the bacterial medium. Cleavage studies revealed a susceptibility of the hydrazone bond in acidic environments. However, a significant excess of acid was required for cleavage to occur, and even at 50 equivalents, it failed to achieve complete release of the subunits. The discrepancy suggests that within these bacterial strains, either there was insufficient uptake of the conjugate species or that it was unable to cleave within the cell lysosome to a sufficient degree. Despite the minimal activity observed towards this Gram-negative *P. aeruginosa*, its activity against *E. coli* suggests that the hydrazone linker provides a facile strategy to broaden the antimicrobial spectrum of novel therapeutics. Upon functionalisation to revitalise the peptide mimic moiety, this linker system will be capable of delivering dual-action conjugates that activate selectively in bacterial lysosomes.

#### 2.3.3. Antimicrobial Activity of Enzyme-Cleavable Linked Conjugate

The cephalosporin conjugate **5** initially underwent direct incubation with bacteria and the enzyme to assess the activity against Gram-positive *S. aureus*. Preincubation with the enzyme was essential to assess antibacterial activity, as the bacterial strains tested do not express *β-*lactamase enzymes. This resulted in a MIC value of >250 µM, which suggested that the conjugate was not sufficiently cleavable, as the peptide mimic subunit in isolation has an MIC of 15.6 µM against this strain. It also indicated that prior to cleavage, the conjugate does not have any bactericidal activity, likely a result of the peptide mimics cationic residue being masked, which prevents interaction with the bacterial cell membrane. To remedy this, HCl was coincubated in the MIC assay, which has been previously reported to effectively facilitate the enzymatic activation of other conjugate systems [35,36]. Upon addition of HCl during preincubation, the MIC of the conjugate was measured as equivalent to the peptide mimic in isolation towards *S. aureus*, suggesting full release of the peptide mimic **1b** to exert its MOA.

The conjugate remained inactive against Gram-negative *E. coli*, which indicated that the spectrum of activity of these conjugates remained restrictive towards Gram-positive strains. As a result, **5** was not tested against *P. aeruginosa*. The lack of enhancement likely resulted from cephalosporin being unable to exert bactericidal effects in a conjugated state, and subsequent inactivation of the cephalosporin portion upon exposure to *β*-lactamase enzymes caused minimal enhancement in specificity towards Gram-negative strains, resulting in activity comparable to peptide mimic **1b** in isolation.

While peptide mimics have been highlighted as a potential solution to the antimicrobial resistance crisis, they have also been limited at times due to off-target interactions with mammalian cells [32,37]. The cephalosporin conjugate provides an interesting strategy to transiently mask cationic residues of toxic peptidomimetics to make effective prodrugs [32,38]. This strategy could be used to revitalise the cytotoxic SMAMPs for the selective targeting of β-lactamase-producing bacteria.

## 3. Materials and Methods

### 3.1. General Notes—Synthesis

All chemical reagents and solvents were purchased from commercial sources (Combi-Blocks (San Diego, CA, USA), Chem Impex (Rajkot, India), Sigma Aldrich (St. Louis, MA, USA), Chem Supply (Gillman, Australia), and Ambeed (Buffalo Grove, IL, USA)) and used without further purification. All reactions were conducted in oven-dried glassware, under an atmosphere of nitrogen or argon as required, unless otherwise stated. Anhydrous solvents were obtained using the PureSolv MD Solvent Purification System (Inert Corporation, Amesbury, MA, USA), by percolation through activated alumina columns. Room temperature refers to the ambient temperature. Yields refer to chromatographically and spectroscopically pure compounds (≥95% purity), unless otherwise stated. Reactions were monitored by TLC using Merk (Darmstadt, Germany) aluminium-backed silica gel 60 F254 plates and visualised under short/long-range UV light and ninhydrin or potassium permanganate stains. Silicycle SiliaFlash 230–400 mesh silica gel 60 (SiliCycle, Quebec City, QC, Canada) was used for manual flash chromatography purification. A CombiFlash system was used for automated flash chromatography. Eluents for flash chromatography are reported as a volume-to-volume ratio of polar:non-polar solvent. ^1^H and ^13^C NMR were recorded on Bruker Avance III HD 300, 400, or 600 spectrometers (Billerica, MA, USA) in the specified solvent. Chemical shifts (δ) are quoted in parts per million (ppm), to the nearest 0.01 ppm, and internally referenced relative to the solvent nuclei. ^1^H NMR spectral data are reported as follows [chemical shift in ppm; multiplicity in br, broad; s, singlet; d, doublet; t, triplet; q, quartet; m, multiplet; or as a combination of these (e.g., dd, dt, etc.)]; coupling constant (*J*) in Hz, integration, proton count, and assignment. HRMS spectra were recorded on a Thermo LTQ Orbitrap XL instrument (Thermo Fisher Scientific, Waltham, MA, USA) by the Bioanalytical Mass Spectrometry Facility at the UNSW Mark Wainwright Analytical Centre. Preparative HPLC was performed on a Shimadzu HPLC instrument (Shimadzu, Kyoto, Japan) using a Grace VisonHT C18 5 µm reverse phase column. A solvent gradient of 5% acetonitrile in milli-Q water (with 0.05% trifluoroacetic acid) to 80% acetonitrile (0.05% trifluoroacetic acid) was used. LCMS was performed on a Shimadzu LCMS 2020 instrument using a solvent gradient of 10–80% acetonitrile in milli-Q water (plus 0.1% formic acid).

#### 3.1.1. General Procedure A for Synthesis of Compounds **2a**, **12**

To a solution of the appropriate amine (1 equiv.) in DMF, EDC (1.5 equiv.), HOBt (1.5 equiv.) and the appropriate carboxylic acid (1 equiv.) were successively added and stirred. DIPEA (2 equiv.) was added dropwise, and the solution was stirred at r.t. for 18 h. After completion, the reaction mixture was diluted with ethyl acetate and washed with 10% (*w*/*v*) citric acid (3×), saturated sodium bicarbonate (3×), and brine (3×). The organic layer was dried over magnesium sulphate and concentrated under reduced pressure. The crude product was purified using flash chromatography with 1:19 ⟶ 3:17 methanol/dichloromethane to afford the pure compound.

#### 3.1.2. General Procedure B for Synthesis of Compounds **2b**, **3b**

The appropriate protected compound (1 mmol) was dissolved in DCM (2 mL) and 4 M HCl in dioxane (2 mL) and allowed to stir at room temperature until TLC indicated complete consumption of starting material. The mixture was then concentrated under reduced pressure and then triturated with diethyl ether to afford the desired compound in sufficient purity.

#### 3.1.3. General Procedure C for Synthesis of Compounds **3a**, **13**

HATU (1.5 equiv.) was added to a solution of the appropriate carboxylic acid (1 equiv.) in DMF and was cooled to 0 °C. DIPEA (2 equiv.) was added dropwise to the mixture and allowed to stir for 10 min, after which the appropriate amine (1 equiv.) was added. After completion, the reaction mixture was diluted with ethyl acetate and washed with water (3×) and brine (3×). The organic layer was dried over magnesium sulphate and concentrated under reduced pressure. The crude product was purified using flash chromatography with 1:19 ⟶ 3:17 methanol/dichloromethane to afford the pure compound.

### 3.2. Analytical Data

The analytical data for intermediates **6**–**10**, **11b**, and **1a**–**1b** have been published previously in the literature [14,15,39].

(*S*)-*N*-(2-(2-(2-(2-Naphthamido)-5-bromobenzamido)-3-(1*H*-indol-3-yl)propanamido)ethyl)-7-chloro-1-cyclopropyl-6-fluoro-4-oxo-1,4-dihydroquinoline-3-carboxamide (**2a**)

Synthesised according to general procedure A from compounds **1a** (134 mg, 0.22 mmol) and **11a** (100 mg, 0.22 mmol). The title compound was obtained as a light yellow solid (67 mg, 30%); m.p. 216.9–219.5 °C; ^1^H NMR (400 MHz, DMSO-*d*_6_) δ 12.31 (s, 1H), 10.76 (d, *J* = 2.4 Hz, 1H), 9.72 (t, *J* = 5.3 Hz, 1H), 9.14 (d, *J* = 7.9 Hz, 1H), 8.53 (d, *J* = 9.0 Hz, 1H), 8.44 (s, 1H), 8.35–8.28 (m, 2H), 8.22 (d, *J* = 6.2 Hz, 1H), 8.08 (d, *J* = 2.4 Hz, 1H), 8.06–7.87 (m, 3H), 7.77 (dd, *J* = 8.9, 2.0 Hz, 2H), 7.73–7.67 (m, 1H), 7.70–7.56 (m, 3H), 7.26 (t, *J* = 8.0 Hz, 1H), 7.21 (d, *J* = 2.3 Hz, 1H), 7.05–6.90 (m, 2H), 4.80–4.70 (m, 1H), 3.65–3.54 (m, 1H), 3.51–3.37 (m, 3H), 3.32–3.11 (m, 3H), 1.25–1.14 (m, 2H), 0.99–0.81 (m, 2H); ^13^C NMR (101 MHz, DMSO-*d*_6_) δ 174.5, 174.4, 171.8, 167.8, 164.8, 164.4, 156.1, 153.6, 147.8, 139.1, 137.9, 137.9, 136.5, 135.2, 134.8, 132.5, 131.8, 131.5, 129.6, 129.0, 128.6, 128.2, 128.1, 127.7, 127.4, 127.3, 127.2, 126.1, 125.9, 124.0, 123.5, 122.4, 122.3, 121.4, 120.5, 118.8, 118.7, 114.9, 112.4, 112.2, 111.8, 111.1, 111.0, 55.2, 39.2, 38.7, 35.7, 28.7, 27.5, 8.0, 7.9; ^19^F NMR (376 MHz, DMSO-*d*_6_) δ −119.49 (dd, *J* = 9.2, 6.3 Hz); HRMS (+ ESI): found *m*/*z*, 883.1401 [M+Na]^+^ C_44_H_35_BrClFN_6_O_5_Na^+^ required 883.1417.

*tert*-Butyl (*S*)-4-(3-((2-(2-(2-(2-naphthamido)-5-bromobenzamido)-3-(1*H*-indol-3-yl)propanamido)ethyl)carbamoyl)-1-cyclopropyl-6-fluoro-4-oxo-1,4-dihydroquinolin-7-yl)piperazine-1-carboxylate (**12**)

Synthesised according to general procedure A from **1a** (200 mg, 0.33 mmol) and **11b** (144 mg, 0.33 mmol). The product was isolated as a white solid (71 mg, 21%); m.p. 216.9–219.5 °C; ^1^H NMR (400 MHz, DMSO-*d*_6_) δ 12.27 (s, 1H), 10.76 (d, *J* = 2.4 Hz, 1H), 9.95 (t, *J* = 5.6 Hz, 1H), 9.18 (d, *J* = 8.0 Hz, 1H), 8.53 (d, *J* = 9.2 Hz, 1H), 8.44 (s, 1H), 8.40–8.28 (m, 2H), 8.12–7.94 (m, 4H), 7.82–7.74 (m, 1H), 7.74–7.52 (m, 5H), 7.39 (d, *J* = 7.5 Hz, 1H), 7.32–7.17 (m, 2H), 7.06–6.90 (m, 2H), 4.82–4.70 (m, 1H), 3.62–3.51 (m, 5H), 3.50–3.36 (m, 2H), 3.31–3.25 (m, 1H), 3.24–3.16 (m, 5H), 2.74 (s, 2H), 1.45 (s, 9H), 1.26–1.13 (m, 2H), 0.95–0.87 (m, 2H); ^13^C NMR (101 MHz, DMSO-*d*_6_) δ 174.6, 171.7, 167.8, 165.0, 164.9, 154.2, 146.8, 139.1, 138.6, 136.5, 135.2, 134.9, 132.6, 131.9, 131.5, 129.6, 129.0, 128.6, 128.3, 128.1, 127.7, 127.4, 123.9, 123.6, 122.5, 121.4, 118.9, 118.7, 114.9, 111. 8, 111.1, 110.5, 106.7, 79.6, 55.2, 50.0, 38.5, 35.3, 28.6, 7.9; ^19^F NMR (376 MHz, DMSO-*d*_6_) δ −124.04–−124.14 (m); HRMS (+ ESI): found *m*/*z*, 1011.3204 [M+H]^+^ C_53_H_53_BrFN_8_O_7_^+^ required 1011.3199.

(*S*)-*N*-(2-(2-(2-(2-Naphthamido)-5-bromobenzamido)-3-(1*H*-indol-3-yl)propanamido)ethyl)-1-cyclopropyl-6-fluoro-4-oxo-7-(piperazin-1-yl)-1,4-dihydroquinoline-3-carboxamide (**2b**)

Synthesised according to general procedure B from 12 (60 mg, 0.06 mmol). The title compound was isolated as a yellow solid (55 mg, 97%); m.p. 213.6–219.5 °C; ^1^H NMR (400 MHz, DMSO-*d*_6_) δ 12.28 (s, 1H), 10.78 (d, *J* = 2.5 Hz, 1H), 9.92 (t, *J* = 5.6 Hz, 1H), 9.53 (s, 2H, NH_2_^+^), 9.17 (d, *J* = 8.0 Hz, 1H), 8.57–8.48 (m, 1H), 8.44 (s, 1H), 8.34 (t, *J* = 3.3 Hz, 2H), 8.07 (d, *J* = 2.8 Hz, 1H), 8.06–7.93 (m, 3H), 7.78 (dd, *J* = 8.6, 1.9 Hz, 1H), 7.74–7.56 (m, 5H), 7.40 (d, *J* = 7.4 Hz, 1H), 7.29–7.18 (m, 2H), 7.06–6.88 (m, 2H), 4.86–4.59 (m, 1H), 3.62–3.42 (m, 5H), 3.49–3.31 (m, 2H), 3.35–3.25 (m, 6H), 2.77–2.67 (m, 2H), 1.25–1.16 (m, 2H), 0.95–0.87 (m, 2H); ^13^C NMR (101 MHz, DMSO) δ 174.5, 171.8, 167.8, 164.9, 164.8, 161.4, 154.1, 151.6, 148.6, 146.9, 143.5, 143.4, 139.1, 138.6, 136.5, 135.2, 134.8, 132.5, 131.9, 131.6, 129.6, 129.0, 128.6, 128.3, 128.1, 127.7, 127.4, 123.9, 123.6, 123.0, 122.8, 122.4, 122.3, 121.9, 121.8, 121.4, 118.9, 118.6, 114.9, 111.8, 111.7, 111.1, 110.6, 106.7, 55.2, 46.9, 43.0, 38.6, 36.3, 35.4, 27.6, 19.0, 7.9; ^19^F NMR (376 MHz, DMSO-*d*_6_) δ −124.19 (dd, *J* = 12.6, 7.4 Hz); HRMS (+ ESI): found *m*/*z*, 911.2643 [M+H]^+^ C_48_H_45_BrFN_8_O_5_^+^ required 911.2675.

(*S*)-*N*-(4-Bromo-2-((1-(4-(7-chloro-1-cyclopropyl-6-fluoro-4-oxo-1,4-dihydroquinoline-3-carbonyl)piperazin-1-yl)-3-(1*H*-indol-3-yl)-1-oxopropan-2-yl)carbamoyl)phenyl)-2-naphthamide (**3a**)

Synthesised according to general procedure C from **11a** (79 mg, 0.28 mmol) and **1b** (205 mg, 0.28 mmol). The title compound was isolated as a yellow solid (108 mg, 43%); m.p. 194.3–197.9 °C; ^1^H NMR (400 MHz, DMSO-*d*_6_) δ 12.31 (s, 1H), 10.76 (d, *J* = 2.4 Hz, 1H), 9.72 (t, *J* = 5.4 Hz, 1H), 9.17 (d, *J* = 7.9 Hz, 1H), 8.51 (d, *J* = 9.0 Hz, 1H), 8.44 (s, 1H), 8.35–8.28 (m, 2H), 8.22 (d, *J* = 6.2 Hz, 1H), 8.11–7.94 (m, 4H), 7.81–7.54 (m, 6H), 7.29–7.18 (m, 2H), 7.05–6.90 (m, 2H), 4.80–4.70 (m, 1H), 3.69–3.54 (m, 1H), 3.55–3.34 (m, 4H), 3.32–3.11 (m, 4H), 1.25–1.13 (m, 2H), 0.97–0.81 (m, 2H); ^13^C NMR (101 MHz, DMSO-*d*_6_) δ 174.5, 174.4, 171.8, 167.8, 164.8, 164.4, 156.1, 153.6, 147.8, 139.1, 137.9, 137.9, 136.9, 136.5, 135.2, 134.9, 134.8, 132.6, 132.5, 131.8, 131.5, 129.6, 129.0, 128.6, 128.2, 128.1, 127.7, 127.4, 127.3, 127.2, 126.1, 125.9, 124.0, 123.5, 122.3, 122.3, 121.4, 120.5, 118.8, 118.7, 114.9, 112.4, 112.2, 111.8, 111.1, 111.0, 55.2, 39.2, 38.7, 35.7, 28.7, 27.5, 8.0, 7.9; ^19^F NMR (376 MHz, DMSO-*d*_6_) δ −120.34–−120.84 (m); HRMS (+ ESI): found *m*/*z*, 909.1581 [M+Na]^+^ C_46_H_37_BrClFN_6_O_5_Na^+^ required 909.1574.

*tert*-Butyl 4-(3-(4-((2-(2-naphthamido)-5-bromobenzoyl)-*L*-tryptophyl)piperazine-1-carbonyl)-1-cyclopropyl-6-fluoro-4-oxo-1,4-dihydroquinolin-7-yl)piperazine-1-carboxylate (**13**)

Synthesised according to general procedure C from **11b** (121 mg, 0.28 mmol) and **1b** (205 mg, 0.28 mmol). The title compound was isolated as an orange solid (157 mg, 54%); m.p. 194.2–198.9 °C; ^1^H NMR (400 MHz, CDCl_3_) δ 12.19 (s, 1H), 10.14 (t, *J* = 5.7 Hz, 1H), 8.70 (d, *J* = 9.0 Hz, 1H), 8.47 (s, 1H), 8.45–8.36 (m, 2H), 7.98–7.88 (m, 2H), 7.90–7.83 (m, 2H), 7.80 (d, *J* = 13.0 Hz, 1H), 7.75–7.67 (m, 1H), 7.63 (d, *J* = 2.3 Hz, 1H), 7.62–7.47 (m, 3H), 7.40–7.28 (m, 2H), 7.22–7.03 (m, 5H), 5.15–4.85 (m, 1H), 3.72–3.59 (m, 4H), 3.59–3.26 (m, 9H), 3.26–3.13 (m, 4H), 1.53 (s, 9H), 1.25–1.12 (m, 2H), 1.07–0.89 (m, 2H); ^13^C NMR (101 MHz, CDCl_3_) δ 175.1, 171.1, 167.7, 166.6, 165.4, 154.6, 154.5, 152.0, 146.6, 144.8, 144.7, 139.1, 138.2, 136.1, 135.4, 134.9, 132.6, 131.4, 130.0, 129.4, 128.6, 128.3, 127.9, 127.7, 127.5, 126.7, 123.4, 123.4, 122.7, 122.2, 121.5, 119.9, 118.5, 115.3, 112.5, 112.3, 111.3, 110.5, 110.2, 104.8, 104.8, 80.3, 77.4, 77.3, 77.1, 76.7, 54.7, 53.5, 49.8, 41.4, 38.5, 34.8, 31.0, 28.4, 28.0, 14.2, 8.2, 8.1; ^19^F NMR (376 MHz, DMSO-*d*_6_) δ −125.12–−125.39 (m); HRMS (+ ESI): found *m*/*z*, 1059.3176 [M+Na]^+^ C_55_H_54_BrFN_8_O_7_Na^+^ required 1059.3175.

(*S*)-*N*-(4-Bromo-2-((1-(4-(1-cyclopropyl-6-fluoro-4-oxo-7-(piperazin-1-yl)-1,4-dihydroquinoline-3-carbonyl)piperazin-1-yl)-3-(1*H*-indol-3-yl)-1-oxopropan-2-yl)carbamoyl)phenyl)-2-naphthamide (**3b**)

Synthesised according to general procedure B from **13** (100 mg, 0.10 mmol). The title compound was isolated as a beige solid (89 mg, 95%); m.p. 161.8–164.1 °C; ^1^H NMR (400 MHz, DMSO-*d*_6_) δ 12.32 (s, 1H), 10.81 (d, *J* = 2.4 Hz, 1H), 9.93 (t, *J* = 5.5 Hz, 1H), 9.87 (s, 1H), 9.54 (s, 2H), 9.20 (d, *J* = 8.0 Hz, 1H), 8.53 (d, *J* = 8.9 Hz, 1H), 8.43 (s, 1H), 8.41–8.29 (m, 2H), 8.10 (d, *J* = 2.4 Hz, 1H), 8.05–7.90 (m, 3H), 7.83–7.54 (m, 5H), 7.39 (d, *J* = 7.4 Hz, 1H), 7.32–7.18 (m, 2H), 7.05–6.90 (m, 2H), 4.80–4.69 (m, 1H), 3.64–3.55 (m, 1H), 3.55–3.35 (m, 8H), 3.36–3.14 (m, 8H), 1.25–1.15 (m, 2H), 0.94–0.87 (m, 2H); ^13^C NMR (101 MHz, DMSO-*d*_6_) δ 174.5, 171.8, 167.8, 164.9, 164.8, 154.1, 151.6, 146.9, 143.5, 143.4, 139.1, 138.6, 136.5, 135.2, 134.8, 132.5, 131.9, 131.6, 129.6, 129.0, 128.6, 128.3, 128.1, 127.7, 127.5, 123.9, 123.6, 122.4, 122.3, 121.9, 121.8, 121.4, 118.9, 118.7, 114.9, 111.9, 111.8, 111.7, 111.1, 110.6, 106.8, 65.4, 55.2, 46.9, 43.0, 38.6, 35.4, 27.6, 15.6, 7.9; ^19^F NMR (376 MHz, DMSO-*d*_6_) δ −124.08–−124.18 (m); HRMS (+ ESI): found *m*/*z*, 937.2829 [M+H]^+^ C_50_H_47_BrFN_8_O_5_^+^ required 937.2831.

(*S*)-*N*-(4-Bromo-2-((1-hydrazineyl-3-(1*H*-indol-3-yl)-1-oxopropan-2-yl)carbamoyl)phenyl)-2-naphthamide (**14**)

A solution of **8** (200 mg, 0.35 mmol) in methanol (5 mL) was cooled to 0 °C. Hydrazine hydrate (0.07 mL, 1.44 mmol) was added to the solution dropwise. THF was added dropwise to the reaction mixture until full dissolution of the solid, and refluxed until reaction completion. The reaction mixture was concentrated under reduced pressure. The resulting precipitate was triturated with diethyl ether to afford the title compound as a light brown solid (162 mg, 81%); m.p. 212.0–216.1 °C; ^1^H NMR (400 MHz, DMSO-*d*_6_) δ 12.21 (s, 1H), 10.78 (d, *J* = 6.7 Hz, 1H), 9.44 (s, 1H), 9.19–9.12 (m, 1H), 8.63–8.52 (m, 1H), 8.49–8.39 (m, 1H), 8.13–7.99 (m, 4H), 7.91–7.83 (m, 1H), 7.75 (s, 1H), 7.72–7.56 (m, 3H), 7.33–7.18 (m, 2H), 7.01–6.92 (m, 2H), 4.85–4.73 (m, 1H), 4.29 (s, 3H), 3.22–3.04 (m, 2H); ^13^C NMR (101 MHz, DMSO-*d*_6_) δ 170.7, 167.7, 165.1, 138.9, 136.5, 135.3, 134.9, 132.6, 131.7, 129.6, 129.1, 128.7, 128.4, 128.2, 127.7, 127.6, 124.1, 123.7, 121.4, 118.9, 118.7, 115.1, 111.8, 110.8, 53.7, 28.0; HRMS (+ ESI): found *m*/*z* 592.0951 [M+Na]^+^ C_29_H_24_BrN_5_NaO_3_^+^ required 592.0960.

1-Cyclopropyl-6-fluoro-7-(4-(4-formylbenzoyl)piperazin-1-yl)-4-oxo-1,4-dihydroquinoline-3-carboxylic acid (**15**)

Ciprofloxacin (200 mg, 0.56 mmol) was dissolved in anhydrous DCM (26 mL) at 0 °C. Triethylamine (311 µL, 2.21 mmol) was added to the solution and stirred for 15 min. A solution of 4-formylbenzoyl chloride (100 mg, 0.56 mmol) in anhydrous DCM (10 mL) was added dropwise to the suspension over 10 min, then warmed to room temperature and stirred for 18 h. Upon completion, the mixture was diluted with DCM and washed with water and brine. The organic layers were dried over magnesium sulphate and concentrated under reduced pressure. The title compound was obtained as a yellow solid (231 mg, 76%); m.p. 201.3–205.4 °C; ^1^H NMR (400 MHz, DMSO-*d*_6_) δ 10.09 (s, 1H), 8.68 (s, 1H), 8.02 (d, *J* = 8.5 Hz, 2H), 7.94 (d, *J* = 13.2 Hz, 1H), 7.70 (d, *J* = 8.1 Hz, 2H), 7.60 (d, *J* = 7.4 Hz, 1H), 3.87 (s, 2H), 3.86–3.76 (m, 1H), 3.55 (s, 2H), 3.46 (s, 2H), 3.38 (s, 2H), 1.38–1.29 (m, 2H), 1.22–1.14 (m, 2H); ^13^C NMR (101 MHz, DMSO-*d*_6_) δ 193.3, 176.9, 168.6, 166.4, 155.8, 152.2, 148.6, 141.6, 139.6, 137.2, 130.1, 128.20, 119.4, 111.7, 107.2, 49.7, 36.4, 8.1; ^19^F NMR (376 MHz, DMSO-*d*_6_) δ −121.73 (dd, *J* = 13.4, 7.7 Hz); HRMS (+ ESI): found *m*/*z* 486.1433 [M+Na]+ C_25_H_22_FN_3_NaO_5_^+^ required 486.1436.

(*E*)-7-(4-(4-((2-((2-(2-Naphthamido)-5-bromobenzoyl)-*L*-tryptophyl)hydrazineylidene)methyl)benzoyl)piperazin-1-yl)-1-cyclopropyl-6-fluoro-4-oxo-1,4-dihydroquinoline-3-carboxylic acid (**4**)

Aldehyde **15** (65 mg, 0.14 mmol) and hydrazide **14** (80 mg, 0.14 mmol) were dissolved in methanol. Catalytic concentrated HCl(aq) was added, and the mixture was heated to reflux for 30 min. The resulting precipitate was filtered to obtain the desired product (139 mg, 98%); m.p. 217.9–219.8 °C; ^1^H NMR (400 MHz, DMSO-*d*_6_) two rotamers δ 12.29 and 12.15 (s, 1H), 12.00 and 11.68 (s, 1H), 10.86 (d, *J* = 8.6 Hz, 1H), 9.42 (d, *J* = 7.5 Hz) and 9.32 (d, *J* = 7.7 Hz, 1H), 8.79 and 8.67 (s, 2H), 8.60 (d, *J* = 9.0 Hz) and 8.56 (d, *J* = 9.0 Hz, 1H), 8.41 (d, *J* = 15.8 Hz, 1H), 8.24–8.12 (m, 1H), 8.10–7.90 (m, 5H), 7.90–7.82 (m, 1H), 7.83–7.69 (m, 3H), 7.69–7.43 (m, 6H), 7.39–7.18 (m, 1H), 7.13–6.86 (m, 2H), 5.84–5.64 and 4.99–4.79 (m, 1H), 4.09–3.53 (m, 4H), 3.51–3.13 (m, 7H), 1.32 (m, 2H), 1.18 (m, 2H); ^13^C NMR (101 MHz, DMSO) δ 178.4, 176.8, 173.2, 169.1, 168.4, 167.8, 166.4, 165.1, 161.5, 154.7, 152.2, 148.5, 146.9, 145.3, 143.7, 139.6, 139.1, 137.2, 136.6, 135.8, 135.4, 134.9, 132.6, 132.1, 131.7, 131.6, 129.5, 129.2, 129.0, 128.6, 128.3, 128.1, 127.5, 124.3, 123.8, 123.7, 123.0, 122.5, 121.4, 119.4, 118.8, 115.2, 112.0, 111.6, 110.7, 110.5, 107.3, 71.8, 54.7, 51.9, 49.9, 36.4, 31.7, 29.9, 29.5, 29.1, 27.7, 27.0, 8.1; ^19^F NMR (376 MHz, DMSO-*d*_6_) δ −121.64–−121.81 (m); HRMS (+ ESI): found *m/z* 1037.2371, C_54_H_44_BrFN_8_NaO_7_^+^ [M+Na]^+^ required 1037.2393.

4-Methoxybenzyl 3-((4-((2-(2-naphthamido)-5-bromobenzoyl)-*L*-tryptophyl)piperazin-1-yl)methyl)-8-oxo-7-(2-phenylacetamido)-5-thia-1-azabicyclo[4.2.0]oct-2-ene-2-carboxylate (**18**)

NaI (46 mg, 0.30 mmol) was added to a solution of chloro-cephalosporin **16** (120 mg, 0.25 mmol) in acetone and stirred for 2 h. Upon completion, the mixture was diluted with DCM and washed thrice with 5% aqueous sodium thiosulfate. The organic layer was dried over anhydrous magnesium sulphate and concentrated under reduced pressure. The resulting solid was dissolved in DMF. Peptide mimic **1b** (160 mg, 0.26 mmol) and sodium bicarbonate (65 mg, 0.77 mmol) were added, and the resulting reaction mixture was stirred for 18 h. The reaction was then diluted with chloroform and washed thrice with brine. The organic layer was extracted and dried over anhydrous magnesium sulphate, then concentrated under reduced pressure to afford the title compound as a yellow solid (143 mg, 54%); m.p. 168.4–173.7 °C; ^1^H NMR (600 MHz, DMSO-*d*_6_) δ 12.24 (d, *J* = 9.7 Hz, 1H), 10.88 (s, 1H), 9.45–9.39 (m, 1H), 9.11 (dd, *J* = 8.4, 2.5 Hz, 1H), 8.60 (d, *J* = 8.9 Hz, 1H), 8.46 (s, 1H), 8.13 (t, *J* = 2.3 Hz, 1H), 8.10–7.97 (m, 3H), 7.87 (dd, *J* = 8.6, 1.9 Hz, 1H), 7.83–7.78 (m, 1H), 7.70–7.57 (m, 3H), 7.33–7.20 (m, 9H), 7.02 (t, *J* = 7.6 Hz, 1H), 6.99–6.93 (m, 1H), 6.91–6.82 (m, 2H), 5.68–5.60 (m, 1H), 5.24–4.90 (m, 4H), 3.67 (d, *J* = 7.1 Hz, 3H), 3.59–3.40 (m, 6H), 3.25–3.14 (m, 4H), 3.08–2.94 (m, 1H), 2.26–1.79 (m, 4H), 1.29–1.17 (m, 1H); ^13^C NMR (151 MHz, DMSO-*d*_6_) δ 171.4, 169.8, 169.8, 167.4, 165.2, 165.2, 165.0, 162.3, 159.8, 159.8, 138.9, 136.5, 136.3, 135.5, 134.9, 132.6, 132.0, 131.6, 130.8, 130.7, 129.6, 129.6, 129.5, 129.1, 128.7, 128.4, 128.2, 127.8, 127.6, 127.5, 127.4, 127.0, 126.8, 124.9, 124.4, 123.7, 122.9, 122.6, 121.4, 118.9, 118.7, 115.2, 114.2, 111.9, 110.3, 110.2, 67.3, 59.5, 58.3, 58.1, 55.5, 55.5, 50.9, 45.6, 42.1, 41.9, 40.5, 27.7, 26.9; HRMS (+ ESI): found *m*/*z* 1074.2832 [M+H]^+^ C_57_H_53_BrN_7_O_8_S^+^ required 1074.2854.

3-((4-((2-(2-Naphthamido)-5-bromobenzoyl)-*L*-tryptophyl)piperazin-1-yl)methyl)-8-oxo-7-(2-phenylacetamido)-5-thia-1-azabicyclo[4.2.0]oct-2-ene-2-carboxylic acid (**5**)

PMB conjugate **18** (38 mg, 0.035 mmol) in chloroform (2 mL) was cooled to 0 °C. Thioanisole (124 μL, 1 mmol) and trifluoroacetic acid (0.6 mL) were added. The reaction mixture was stirred at 0 °C for 2 h. After reaction completion, diethyl ether (4 mL) was added to precipitate the product. The precipitate was allowed to settle, and the supernatant was decanted. The solids were redissolved in chloroform (2 mL), then precipitated by dropwise addition into diethyl ether (10 mL). The resulting white solid was collected and dried under vacuum to yield the title compound as a white solid (31 mg, 93%); m.p. 163.0–165.2 °C; ^1^H NMR (400 MHz, DMSO-*d*_6_) δ 12.21 (s, 1H), 10.88 (d, *J* = 2.4 Hz, 1H), 9.40 (d, *J* = 7.4 Hz, 1H), 9.09 (d, *J* = 8.4 Hz, 1H), 8.58 (d, *J* = 8.9 Hz, 1H), 8.47 (d, *J* = 1.8 Hz, 1H), 8.19–7.96 (m, 4H), 7.89 (dd, *J* = 8.6, 1.8 Hz, 1H), 7.80 (dd, *J* = 8.9, 2.4 Hz, 1H), 7.71–7.60 (m, 2H), 7.59 (d, *J* = 7.9 Hz, 1H), 7.38–7.16 (m, 7H), 7.08–7.00 (m, 1H), 7.00–6.92 (m, 1H), 5.66–5.58 (m, 1H), 5.18 (q, *J* = 7.4 Hz, 1H), 5.03 (d, *J* = 4.7 Hz, 1H), 3.63–3.40 (m, 6H), 3.27–3.07 (m, 4H), 2.99 (d, *J* = 13.4 Hz, 1H), 2.28 (s, 2H), 2.03 (s, 1H), 1.79 (s, 1H), 1.24 (s, 1H); ^13^C NMR (151 MHz, DMSO-*d*_6_) δ 171.4, 169.9, 167.4, 165.0, 164.9, 163.6, 138.9, 136.5, 136.3, 134.9, 132.7, 132.0, 131.6, 129.6, 129.6, 129.5, 129.1, 128.7, 128.7, 128.4, 128.2, 127.8, 127.6, 127.0, 124.4, 123.7, 122.9, 122.7, 121.4, 118.9, 118.7, 115.2, 112.0, 110.2, 59.4, 58.1, 58.0, 52.5, 50.9, 42.0, 40.5, 27.6, 27.0, 1.6; HRMS (+ ESI): found *m*/*z* 954.2261 [M+H]^+^ C_49_H_45_BrN_7_O_7_ S^+^ required 954.2279.

### 3.3. Preactivation of Compound **5** for MIC Determination

Conjugate **5** (400 μL of 410 μg mL^−1^ in CAHMB) was mixed with HCl (2 equiv.) and β-lactamase enzyme (100 μL) and incubated for 24 h at 37 °C with shaking at 180 rpm. The preactivated compound was subsequently used for biological testing.

### 3.4. Minimum Inhibitory Concentration Broth Microdilution Assay

MIC broth dilution assays were used to assess antibacterial activity. The bacterial strains used were *S. aureus* SA38 (isolated from a corneal infection), *E. coli* K12 (ATCC: 10798; isolated from faeces), and *P. aeruginosa* PAO1 (isolated from a wound in Melbourne, Australia). A single, pure colony of bacteria was cultured overnight in cation-adjusted Mueller-Hinton Broth (CAMHB) at 37 °C with 200 rpm shaking. The resulting culture was centrifuged at 3000× *g* three times, followed by washing with 1x PBS. The culture was then resuspended in CAMHB and diluted to achieve an OD660 of 0.1 (corresponding to 10^8^ CFU/mL). This suspension was then further diluted by 50-fold to a final concentration of 2 × 10^6^ CFU/mL. 100 µL of this inoculation was then added to wells on a 96-well plate (Costar, Arlington, VI, USA) containing 100 µL of serially diluted AMP mimic conjugate. For visibly insoluble compounds, the OD660 was recorded on a FLUOstar Omega plate reader (BMG LABTECH GmbH, Ortenberg, Germany) before incubation, then the plate was sealed with parafilm and incubated overnight at 37 °C with shaking at 200 rpm. Wells containing media only were used as blanks (unless insoluble), while wells with bacteria and media but no compounds were used as negative controls. The MIC was determined as the lowest concentration of compound that had an OD660 within 1% of the blank for the well. Three experiments, each consisting of three technical replicates, were performed. MIC value was determined after three concordant values were obtained.

### 3.5. Hydrazone Cleavage Studies

Compound **4** (10 mg) was dissolved in 500 µL of DMSO*-d*_6_. To the NMR solution, an appropriate equivalence of concentrated HCl was added and mixed. ^1^H NMR (DMSO-*d*_6_) experiments were conducted at 0.5, 1, 2.5, 24, and 72 h intervals after acid addition. Hydrolysis was quantified through the emergence of the formyl peak present at 10.10 ppm, relative to amide proton peaks at 10.86 ppm and 9.42 ppm, each integrating for one proton, respectively.

### 3.6. Enzyme Cleavage Studies

Compound **5** (4.1 mg) was dissolved in 500 µL of DMSO-*d*_6_. To the NMR solution, 20 µL of a 200 units/mL enzyme stock solution prepared from penicillinase from *Bacillus cereus* (Sigma-Aldrich, lyophilized powder, 1500–3000 units/mg protein) was added and mixed. ^1^H NMR (DMSO-*d*_6_) experiments were conducted at 10 min, 20 min, 6 h, 1 day, and 4-day intervals after enzyme addition.

## 4. Conclusions

Within the presented work, a library of antibiotic–peptidomimetic conjugates, composed of either non-cleavable amide, acid-labile hydrazone, or enzyme-labile cephalosporin linker systems, was synthesised and their biological activity was assessed. Among the synthesised structures, four of the conjugate systems had activity towards Gram-negative bacterial strains. The non-cleavable amide conjugate class was modulated between chloro- and piperazine-substituted fluoroquinolones, as well as primary and secondary amines. The non-cleavable primary amide conjugate between the peptidomimetic and ciprofloxacin had the greatest Gram-negative activity towards *E. coli* and *P. aeruginosa* with MICs of 7.8 µM and 15.6 µM, respectively. The hydrazone conjugate had high activity in both *S. aureus* and *E. coli*. Cleavage studies suggested moderate release, with the greatest hydrolysis observed at 50 equivalents of HCl. Despite the acid dependence, full release of the formylated ciprofloxacin was not observed, indicating stability at the linkage site. The enzymatically labile linker retained activity towards *S. aureus* but was inactive against Gram-negative strains. Cleavage studies suggested rapid cleavage of the linker, highlighting its ability to selectively treat β-lactamase-expressing strains. This work highlights the viability of conjugation as a strategy to enhance antimicrobial properties and spectrum of activity for peptidomimetics and antibiotics to overcome the mounting antimicrobial resistance crisis.

## Figures and Tables

**Table 1:** Hydrolysis of conjugate **4** after the addition of acid.

	% Hydrolysed		
Time (h)	10 Equiv.	30 Equiv.	50 Equiv.
0.5	9	28	29
1	12	30	35
2.5	14	33	39
24	17	34	40
72	23	35	44

**Table 2:** MIC of synthesised conjugates and their anticipated cleaved products in isolation.

		MIC (µM)		
Compound	Role	*S. aureus* SA38	*E. coli* K12	*P. aeruginosa* PAO1
Peptide mimic				
**1a**	SMAMP subunit	3.9	>250	>250
**1b**	SMAMP subunit	15.6	>250	>250
Non-cleavable linker				
**2a**	Conjugate	>250	>250	31.25
**2b**	Conjugate	15.6	7.8	15.6
**3a**	Conjugate	>250	>250	>250
**3b**	Conjugate	7.8	7.8	125
Acid cleavable linker				
**14**	Anticipated product	>250	>250	>250
**15**	Anticipated product	7.8	7.8	31.3
**4**	Conjugate	7.8	15.6	>250
Enzyme cleavable linker				
**5**	Conjugate	>250	>250	>250
**5 + Penicillinase** *	Conjugate + Enzyme	15.6	>250	NT
**1b**	Anticipated product	15.6	>250	>250

NT = Not tested. * Treatment of conjugate with the enzyme during preincubation.

## Data Availability

Data is contained within the article and Supplementary Materials.

## Supplementary Materials

The following supporting information can be downloaded at: https://www.mdpi.com/article/10.3390/antibiotics15050484/s1. File S1: ^1^H NMR and ^13^C NMR data, LCMS trace.
