# Supplementary material for: Conjugation of Antibiotics to Peptidomimetics Enhances Antimicrobial Spectrum of Activity
Source: Antibiotics (Basel). 2026 May 11;15(5):484. doi: 10.3390/antibiotics15050484 (PMC13203689; doi:10.3390/antibiotics15050484)
Supplement: Supplementary file 1 [file antibiotics-15-00484-s001.zip › antibiotics-4302271-supplementary.pdf]

(S)-N-(2-(2-(2-(2-naphthamido)-5-bromobenzamido)-3-(1H-indol-3-yl)propanamido)ethyl)-7-chloro-1-cyclopropyl-6-fluoro-4-oxo-1,4-dihydroquinoline-3-carboxamide (2a)

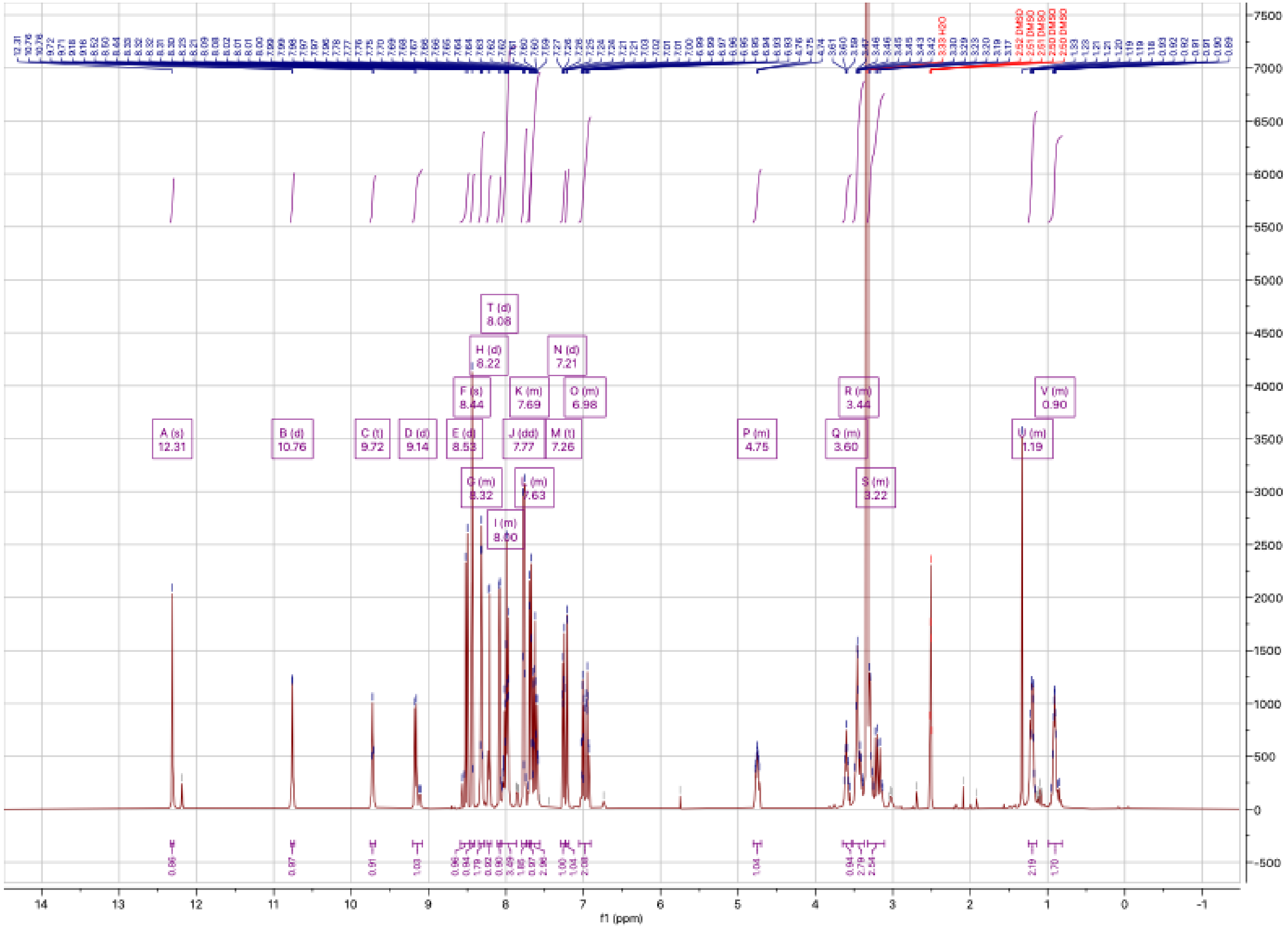

(S)-N-(2-(2-(2-(2-naphthamido)-5-bromobenzamido)-3-(1H-indol-3-yl)propanamido)ethyl)-7-chloro-1-cyclopropyl-6-fluoro-4-oxo-1,4-dihydroquinoline-3-carboxamide (**2a**)

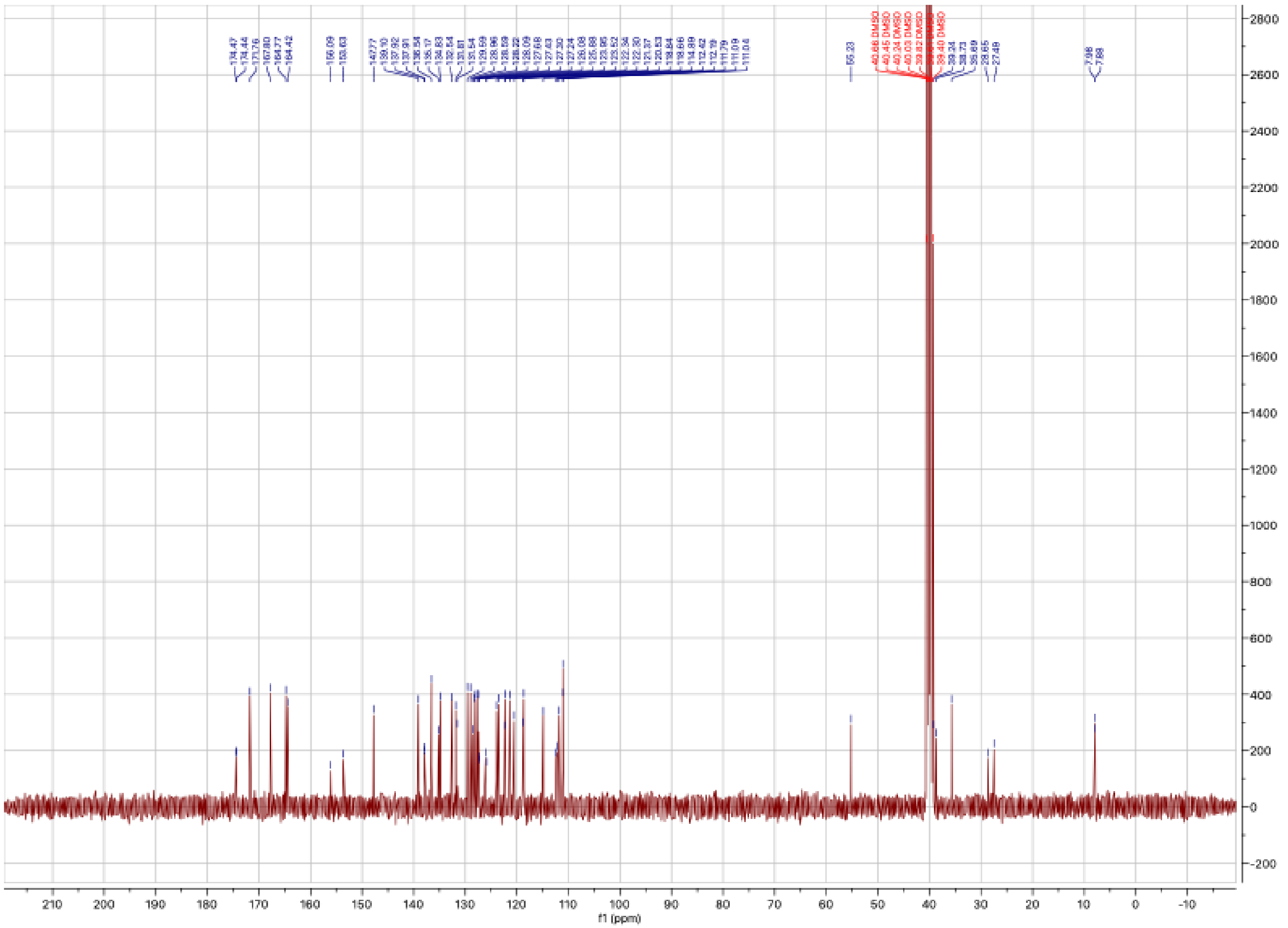

tert-butyl (S)-4-(3-((2-(2-(2-(2-naphthamido)-5-bromobenzamido)-3-(1H-indol-3-yl)propanamido)ethyl)carbamoyl)-1-cyclopropyl-6-fluoro-4-oxo-1,4-dihydroquinolin-7-yl)piperazine-1-carboxylate (**12**)

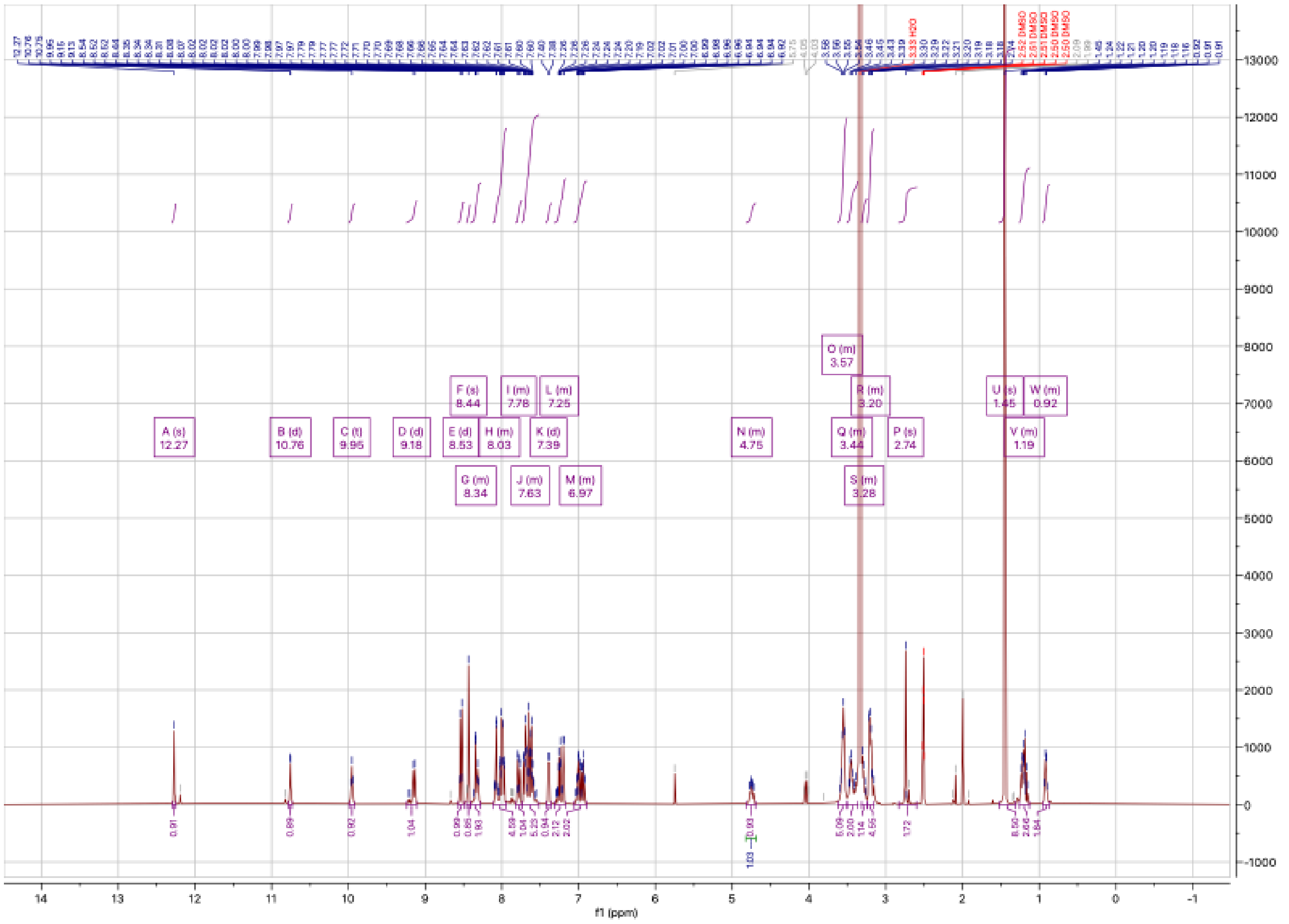

naphthamido)-5-bromobenzamido)-3-(1H-indol-3-yl)propanamido)ethyl)carbamoyl)-1-cyclopropyl-6-fluoro-4-oxo-1,4-dihydroquinolin-7-yl)piperazine-1-carboxylate (**12**)

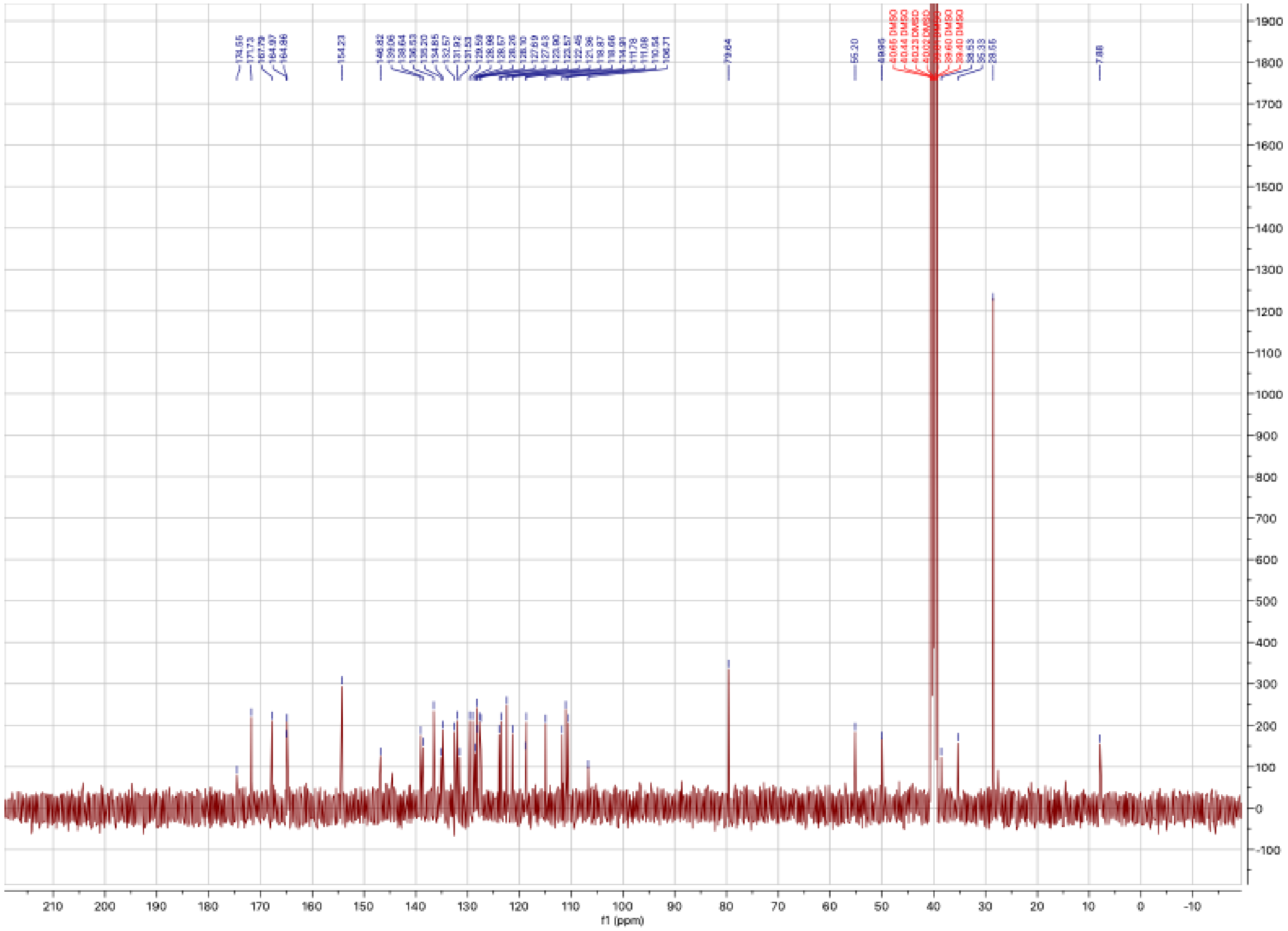

(S)-N-(2-(2-(2-(2-naphthamido)-5-bromobenzamido)-3-(1H-indol-3-yl)propanamido)ethyl)-1-cyclopropyl-6-fluoro-4-oxo-7-(piperazin-1-yl)-1,4-dihydroquinoline-3-carboxamide (**2b**)

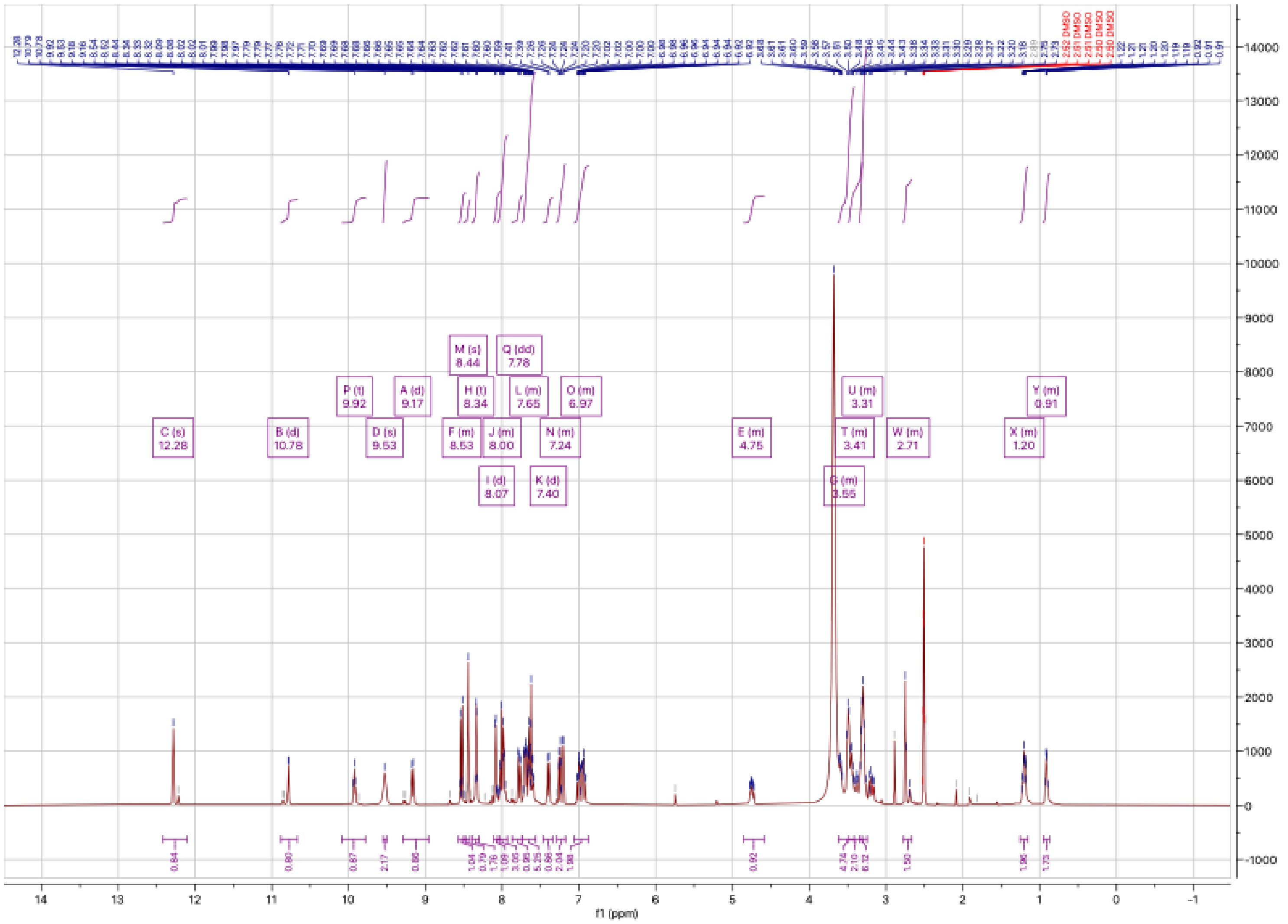

(S)-N-(2-(2-(2-(2-naphthamido)-5-bromobenzamido)-3-(1H-indol-3-yl)propanamido)ethyl)-1-cyclopropyl-6-fluoro-4-oxo-7-(piperazin-1-yl)-1,4-dihydroquinoline-3-carboxamide (**2b**)

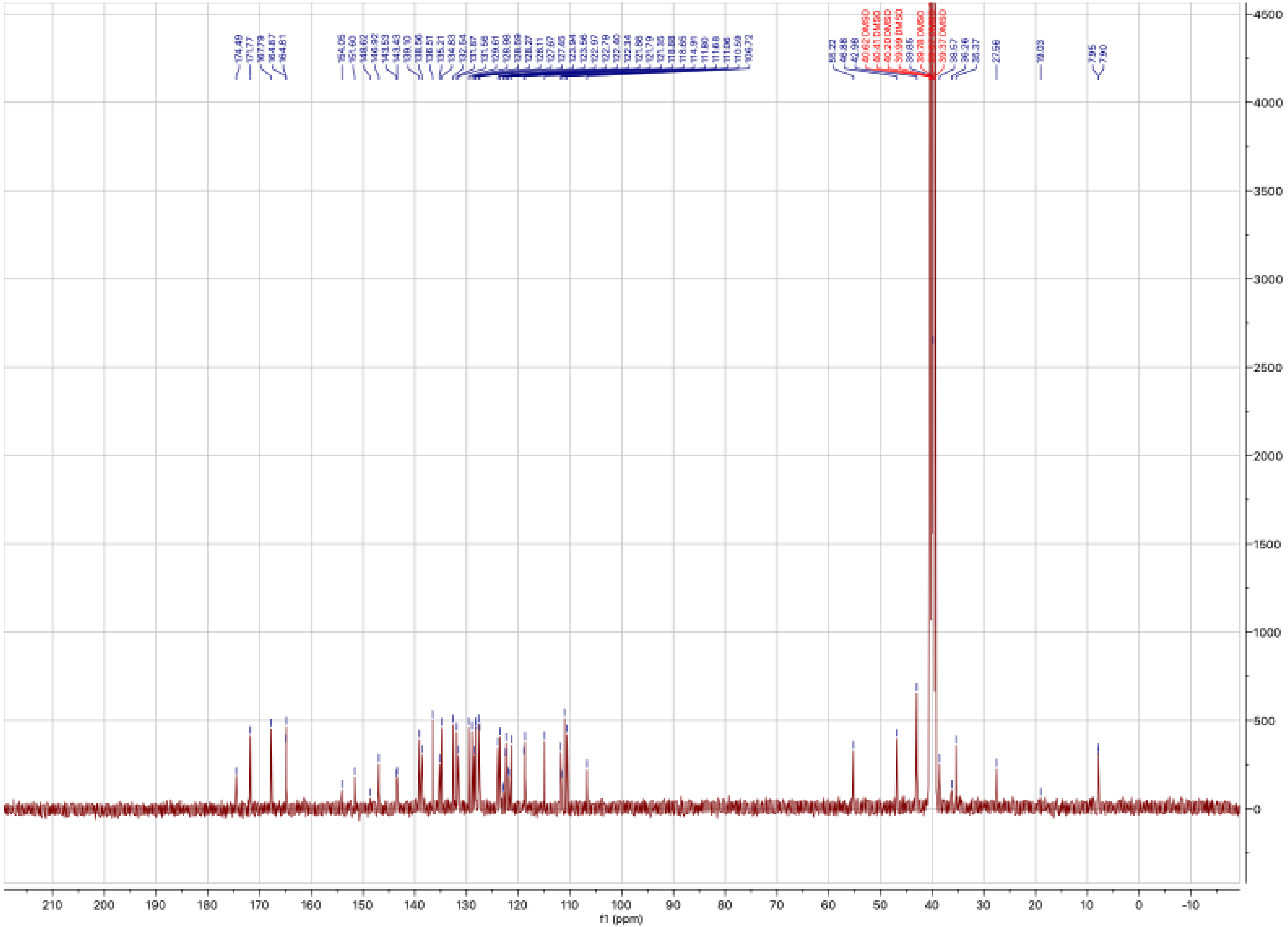

(S)-N-(4-bromo-2-((1-(4-(7-chloro-1-cyclopropyl-6-fluoro-4-oxo-1,4-dihydroquinoline-3-carbonyl)piperazin-1-yl)-3-(1H-indol-3-yl)-1-oxopropan-2-yl)carbamoyl)phenyl)-2-naphthamide (**3a**)

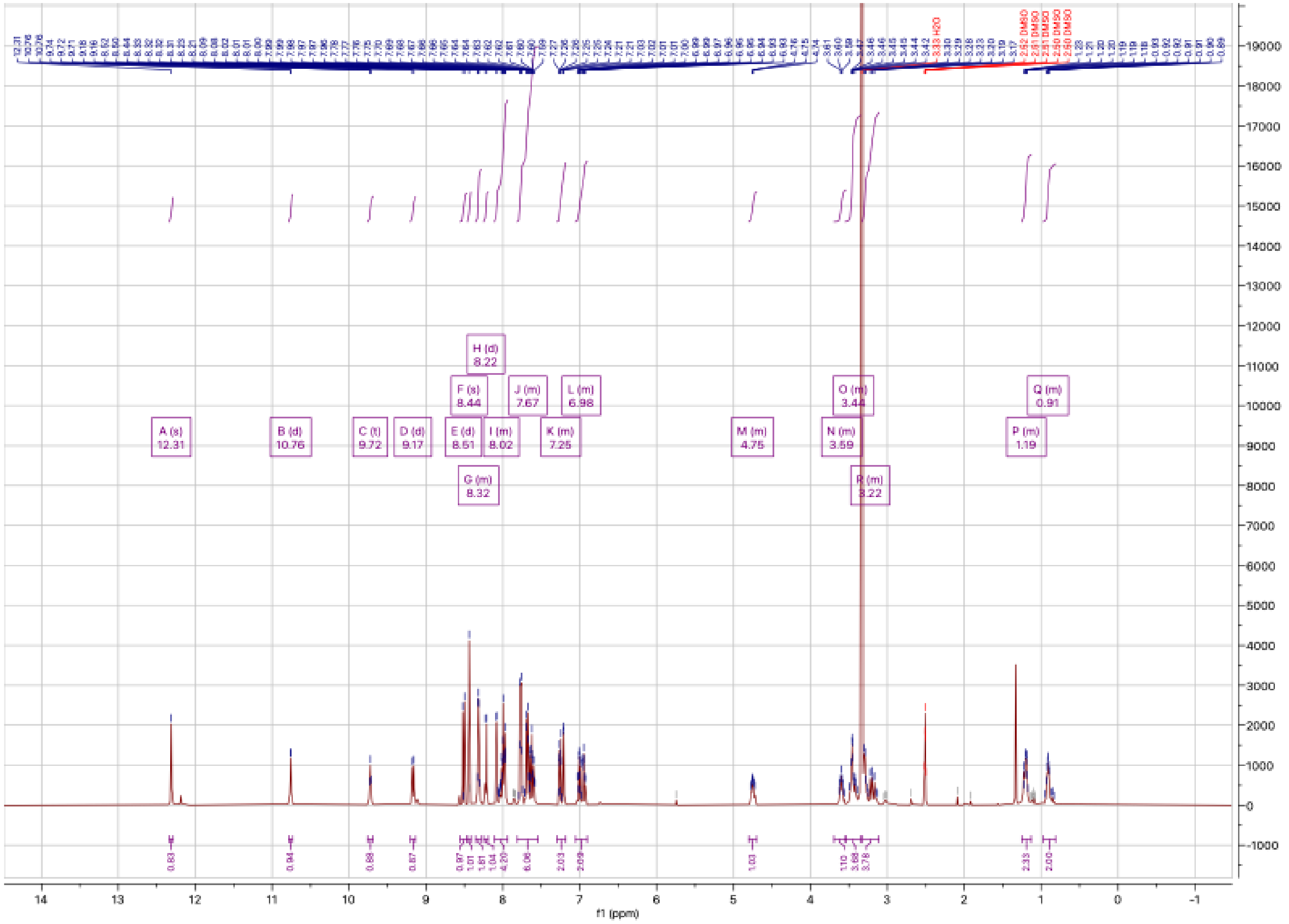

(S)-N-(4-bromo-2-((1-(4-(7-chloro-1-cyclopropyl-6-fluoro-4-oxo-1,4-dihydroquinoline-3-carbonyl)piperazin-1-yl)-3-(1H-indol-3-yl)-1-oxopropan-2-yl)carbamoyl)phenyl)-2-naphthamide (**3a**)

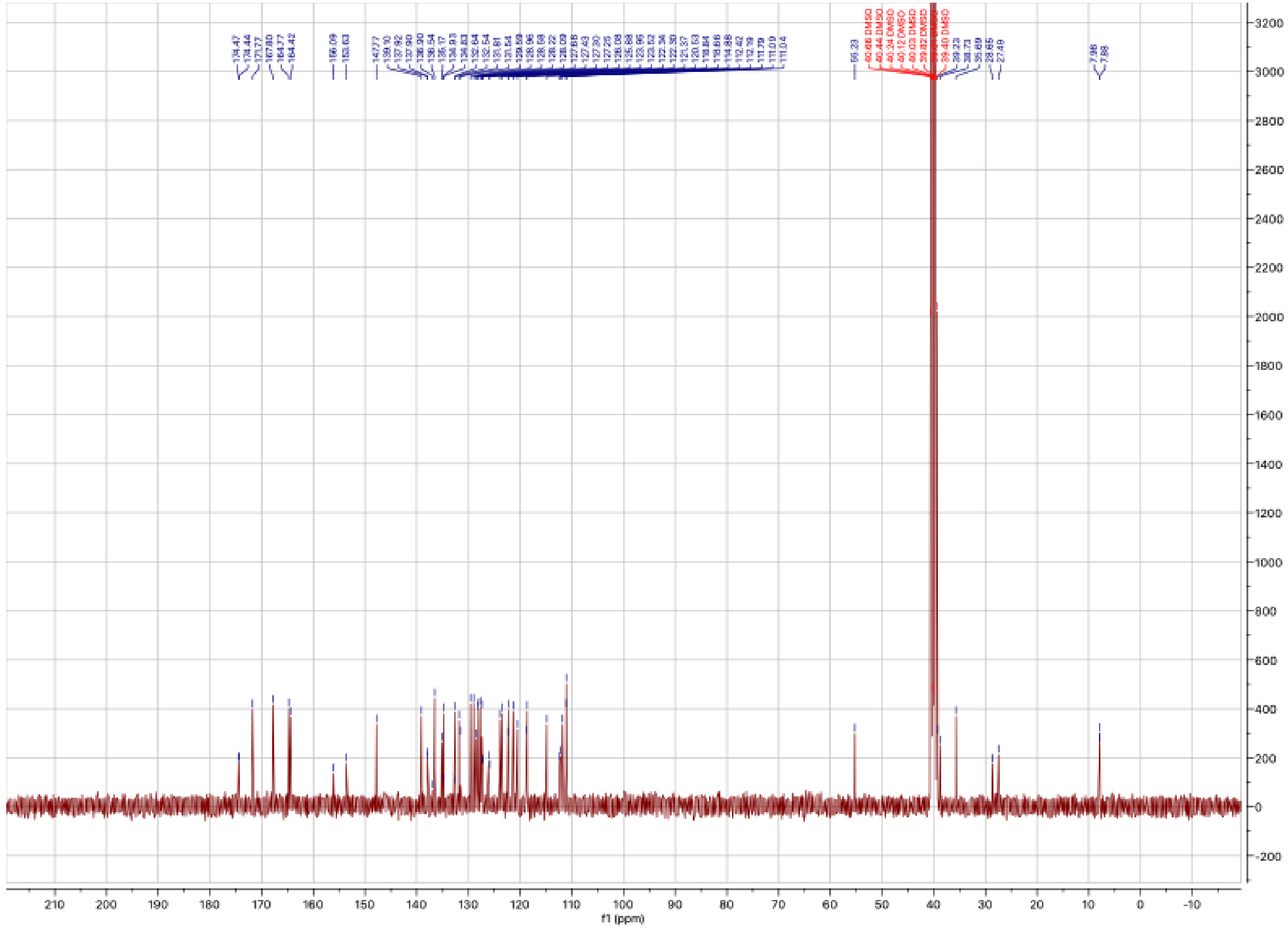

tert-butyl 4-(3-(4-((2-(2-naphthamido)-5-bromobenzoyl)-L-tryptophyl)piperazine-1-carbonyl)-1-cyclopropyl-6-fluoro-4-oxo-1,4-dihydroquinolin-7-yl)piperazine-1-carboxylate (**13**)

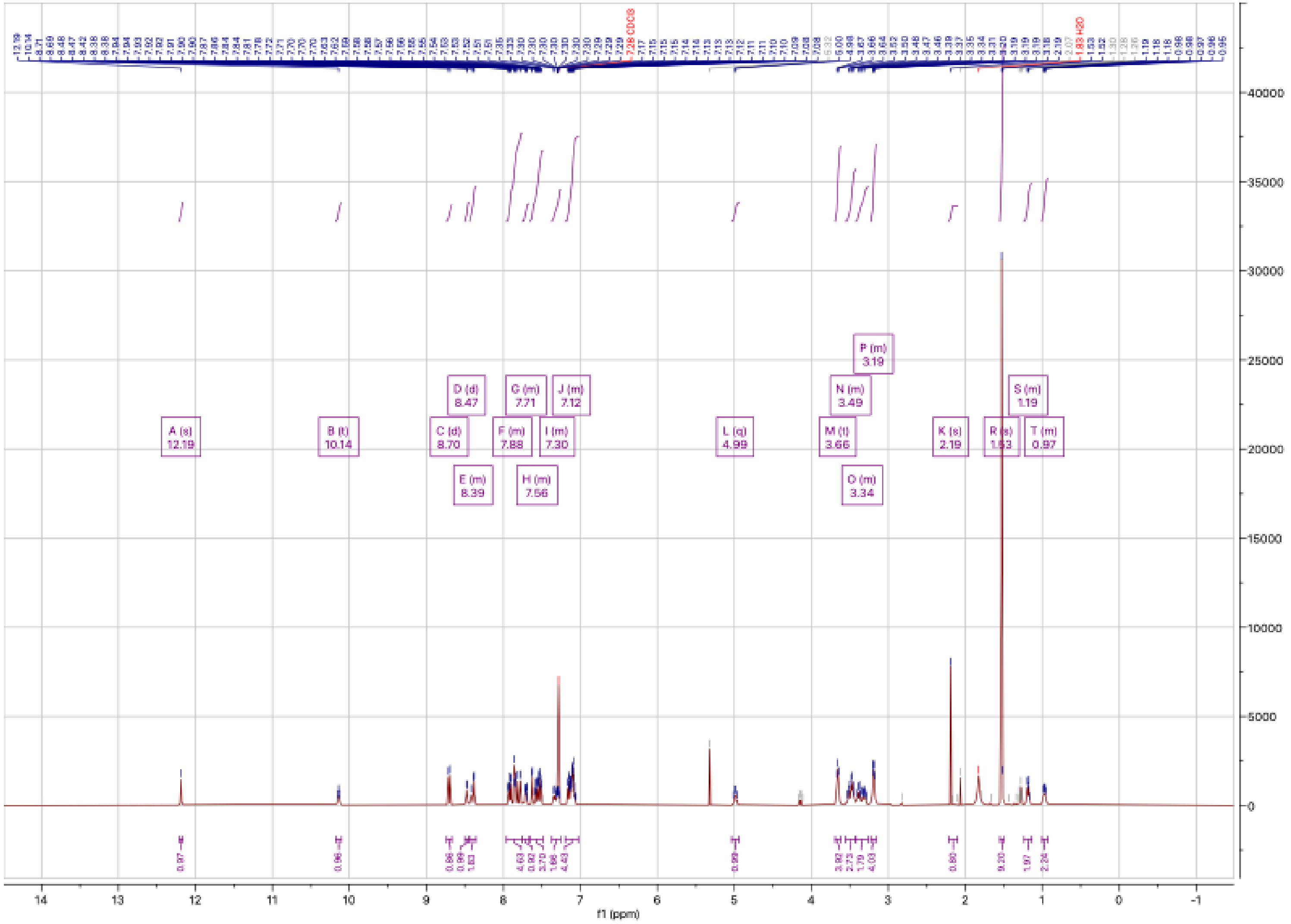

tert-butyl 4-(3-(4-((2-(2-naphthamido)-5-bromobenzoyl)-L-tryptophyl)piperazine-1-carbonyl)-1-cyclopropyl-6-fluoro-4-oxo-1,4-dihydroquinolin-7-yl)piperazine-1-carboxylate (**13**)

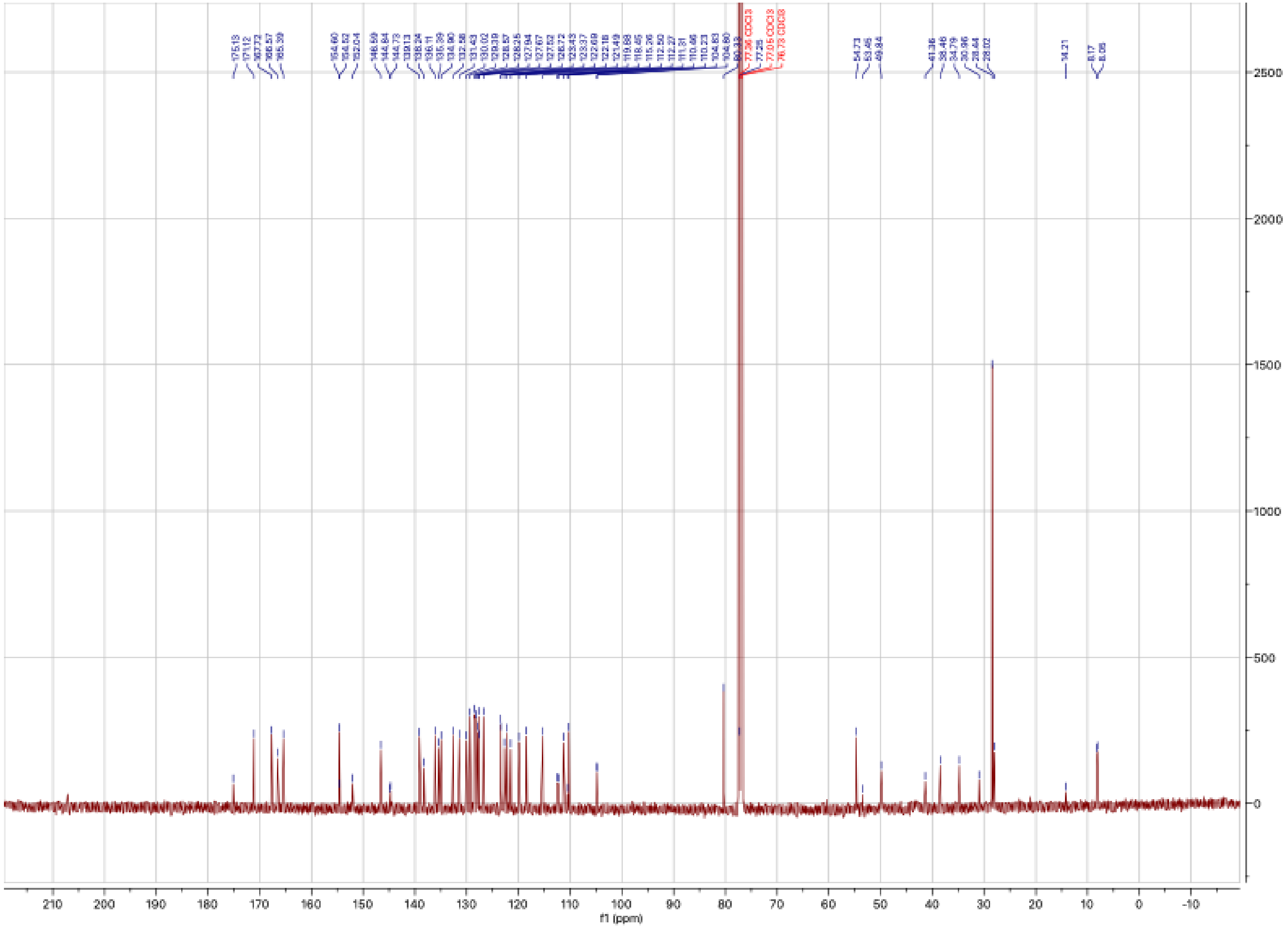

(S)-N-(4-bromo-2-((1-(4-(1-cyclopropyl-6-fluoro-4-oxo-7-(piperazin-1-yl)-1,4-dihydroquinoline-3-carbonyl)piperazin-1-yl)-3-(1H-indol-3-yl)-1-oxopropan-2-yl)carbamoyl)phenyl)-2-naphthamide (**3b**)

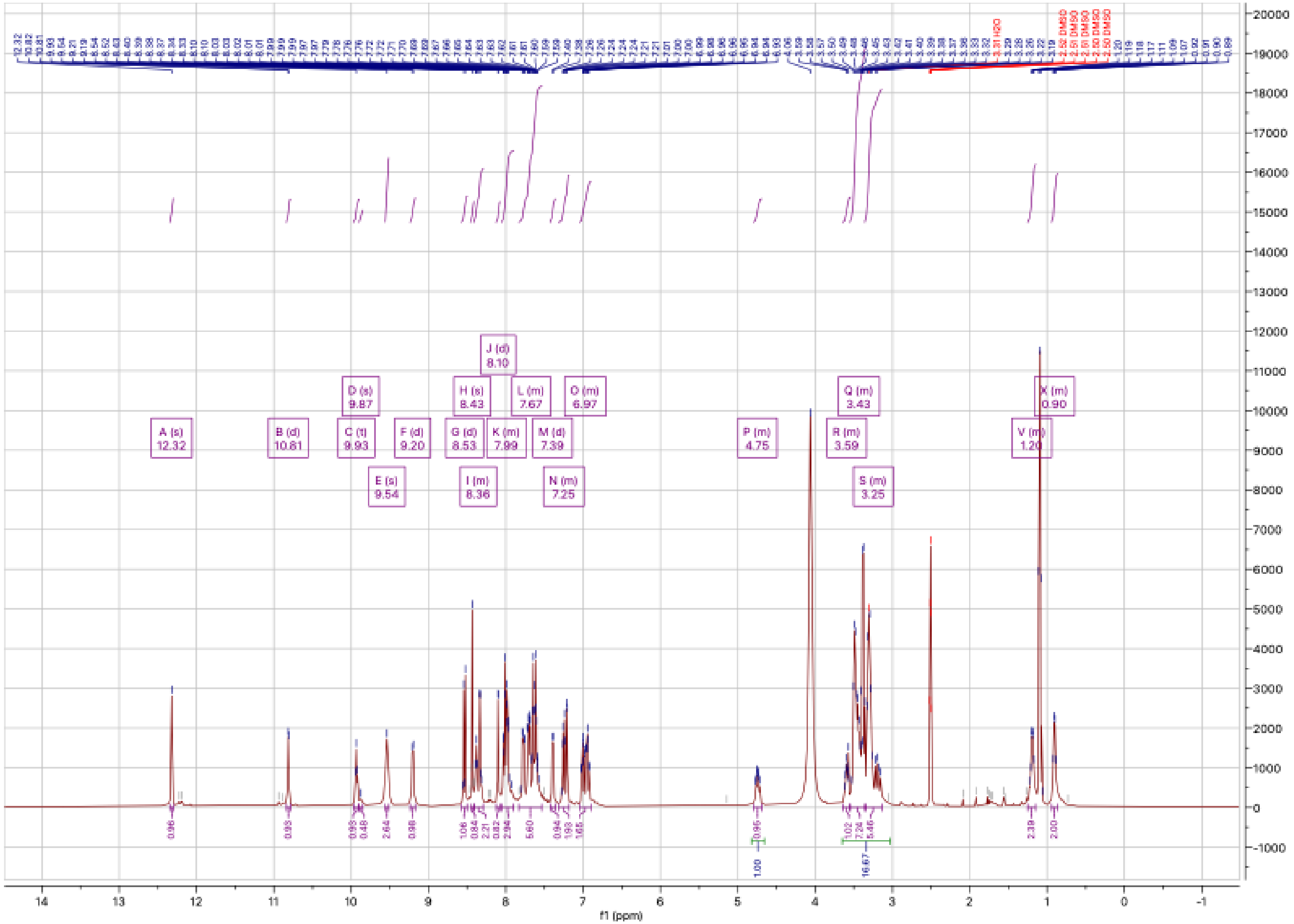

(S)-N-(4-bromo-2-((1-(4-(1-cyclopropyl-6-fluoro-4-oxo-7-(piperazin-1-yl)-1,4-dihydroquinoline-3-carbonyl)piperazin-1-yl)-3-(1H-indol-3-yl)-1-oxopropan-2-yl)carbamoyl)phenyl)-2-naphthamide (**3b**)

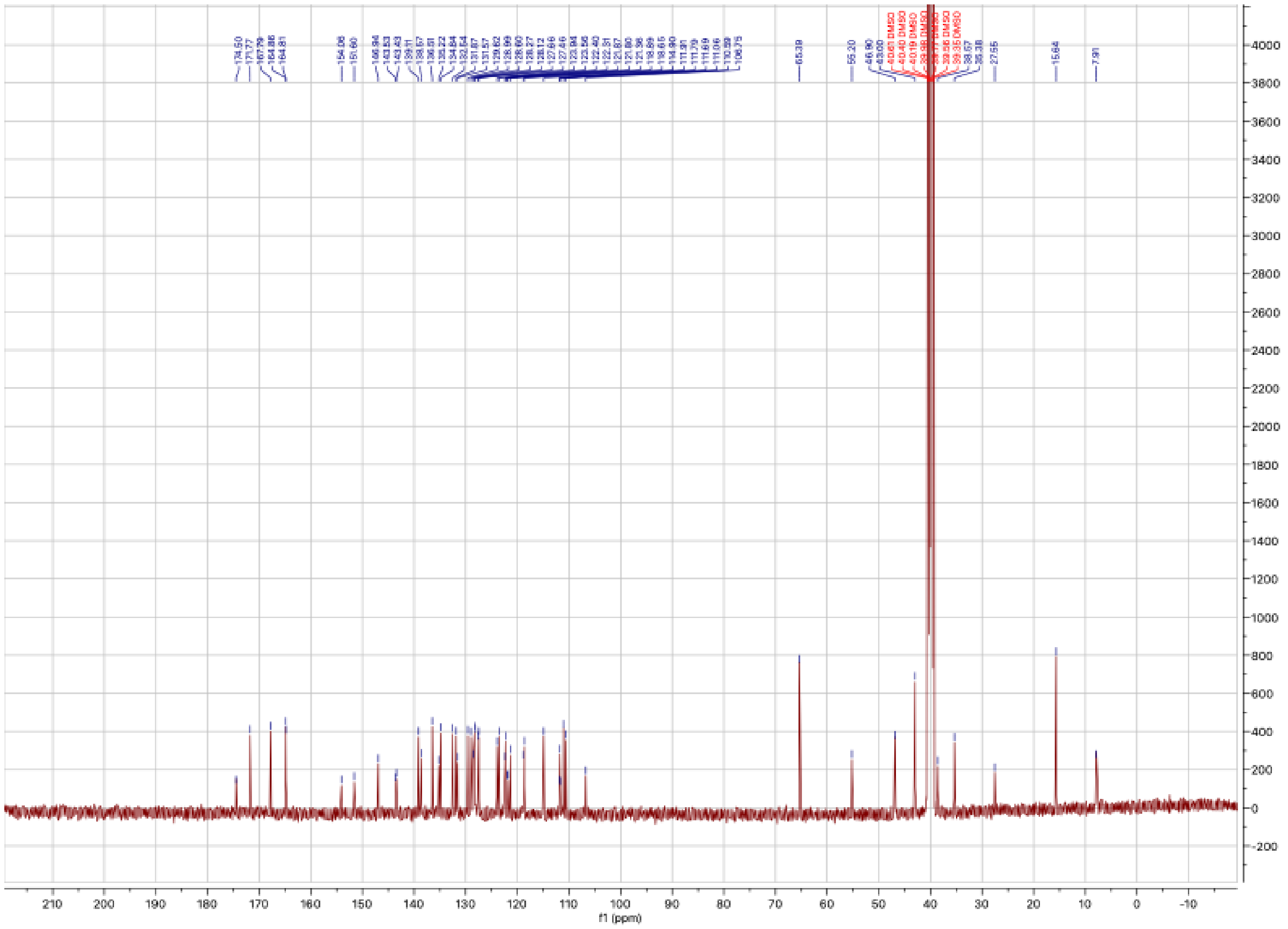

(S)-N-(4-bromo-2-((1-hydrazineyl-3-(1H-indol-3-yl)-1-oxopropan-2-yl)carbamoyl)phenyl)-2-naphthamide (**14**)

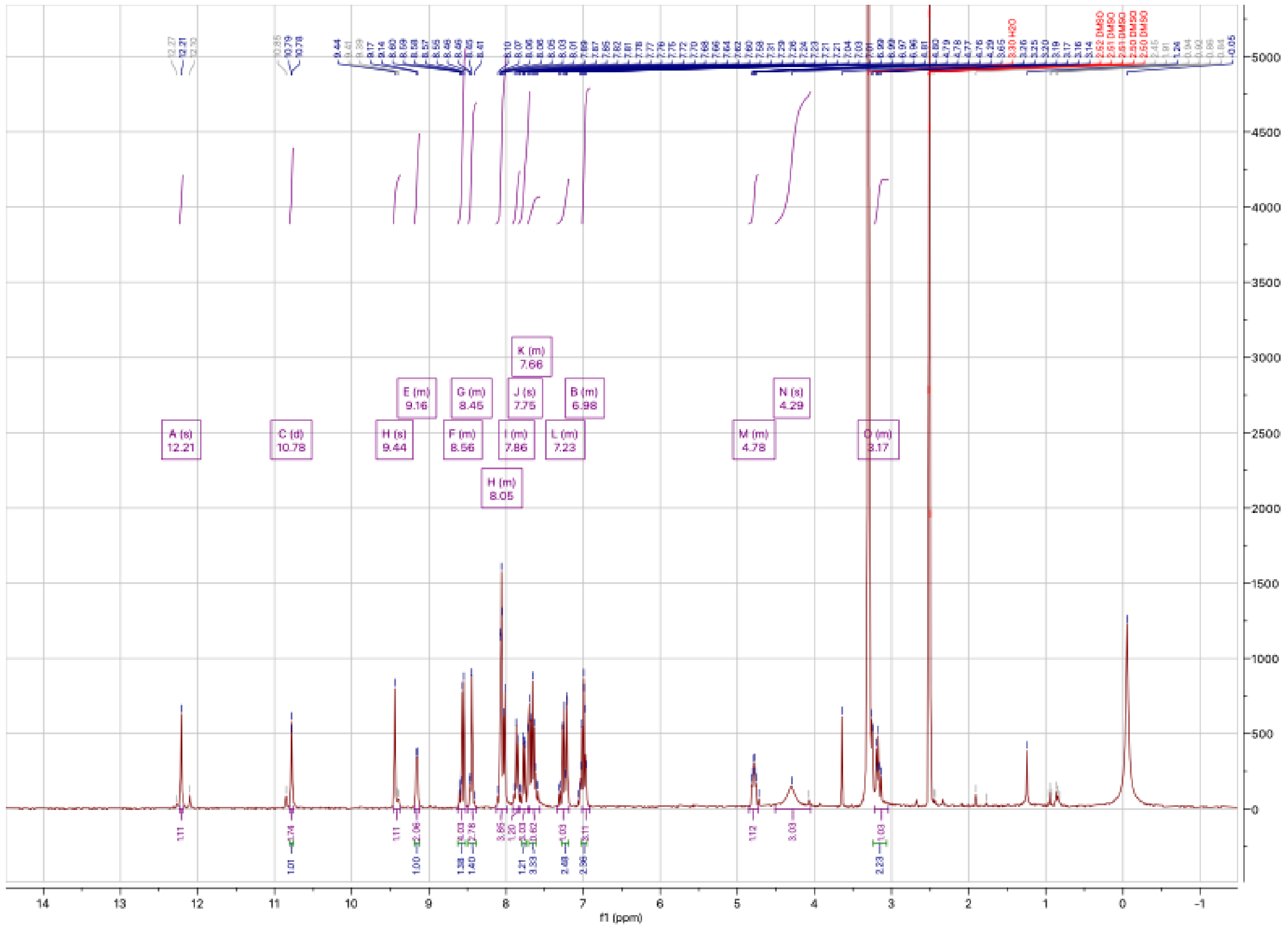

(S)-N-(4-bromo-2-((1-hydrazineyl-3-(1H-indol-3-yl)-1-oxopropan-2-yl)carbamoyl)phenyl)-2-naphthamide (**14**)

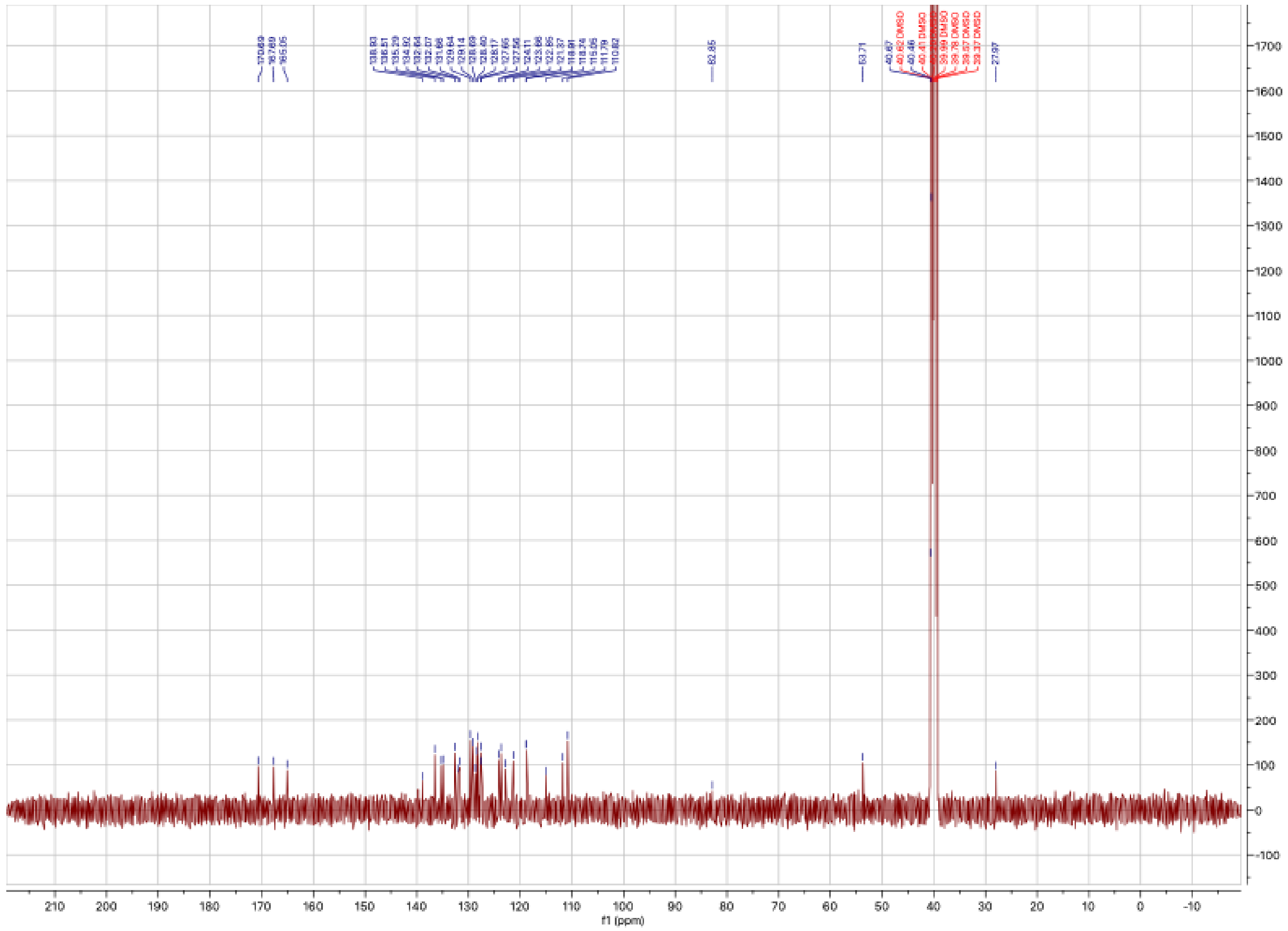

1-cyclopropyl-6-fluoro-7-(4-(4-formylbenzoyl)piperazin-1-yl)-4-oxo-1,4-dihydroquinoline-3-carboxylic acid (**15**)

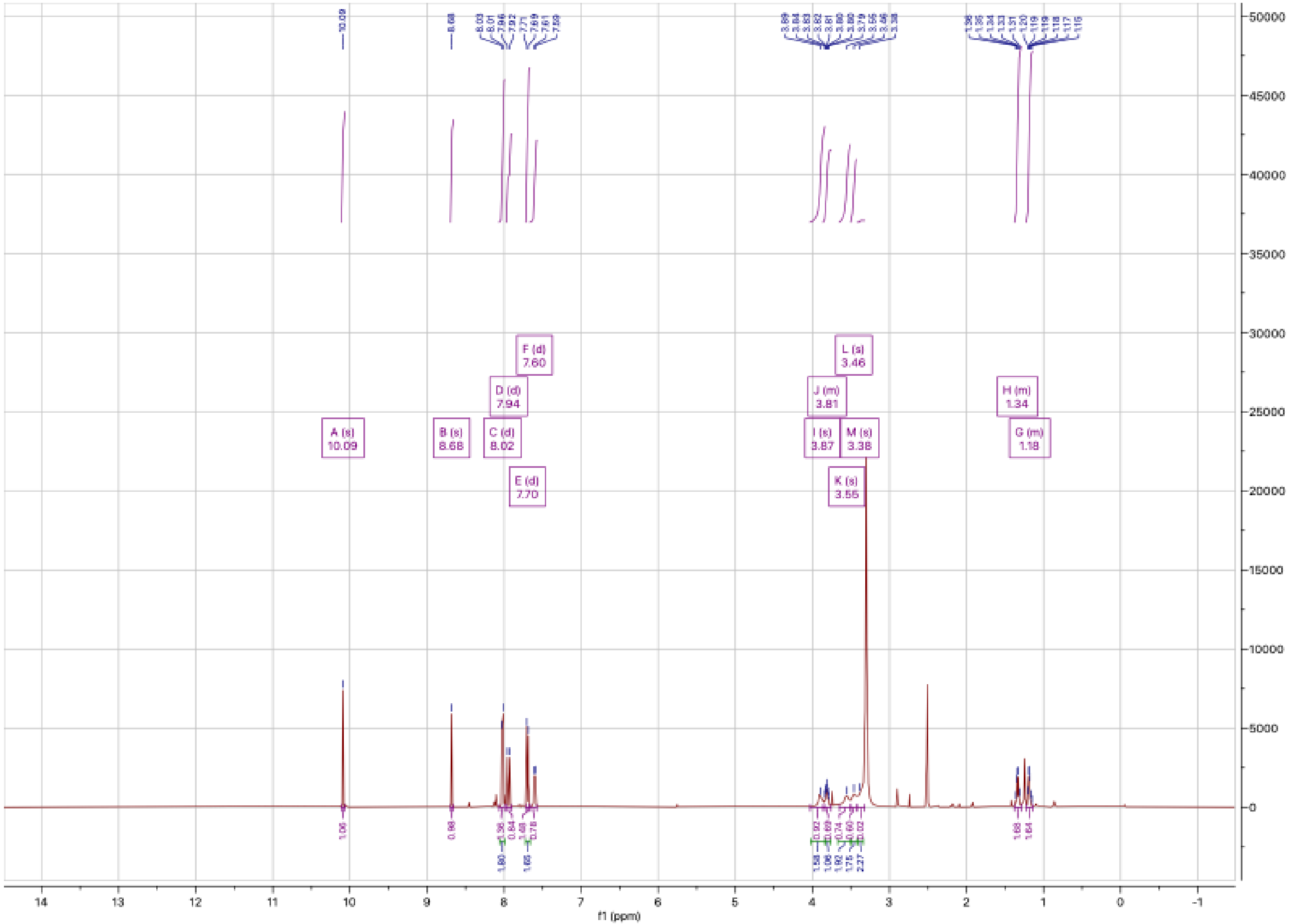

1-cyclopropyl-6-fluoro-7-(4-(4-formylbenzoyl)piperazin-1-yl)-4-oxo-1,4-dihydroquinoline-3-carboxylic acid (**15**)

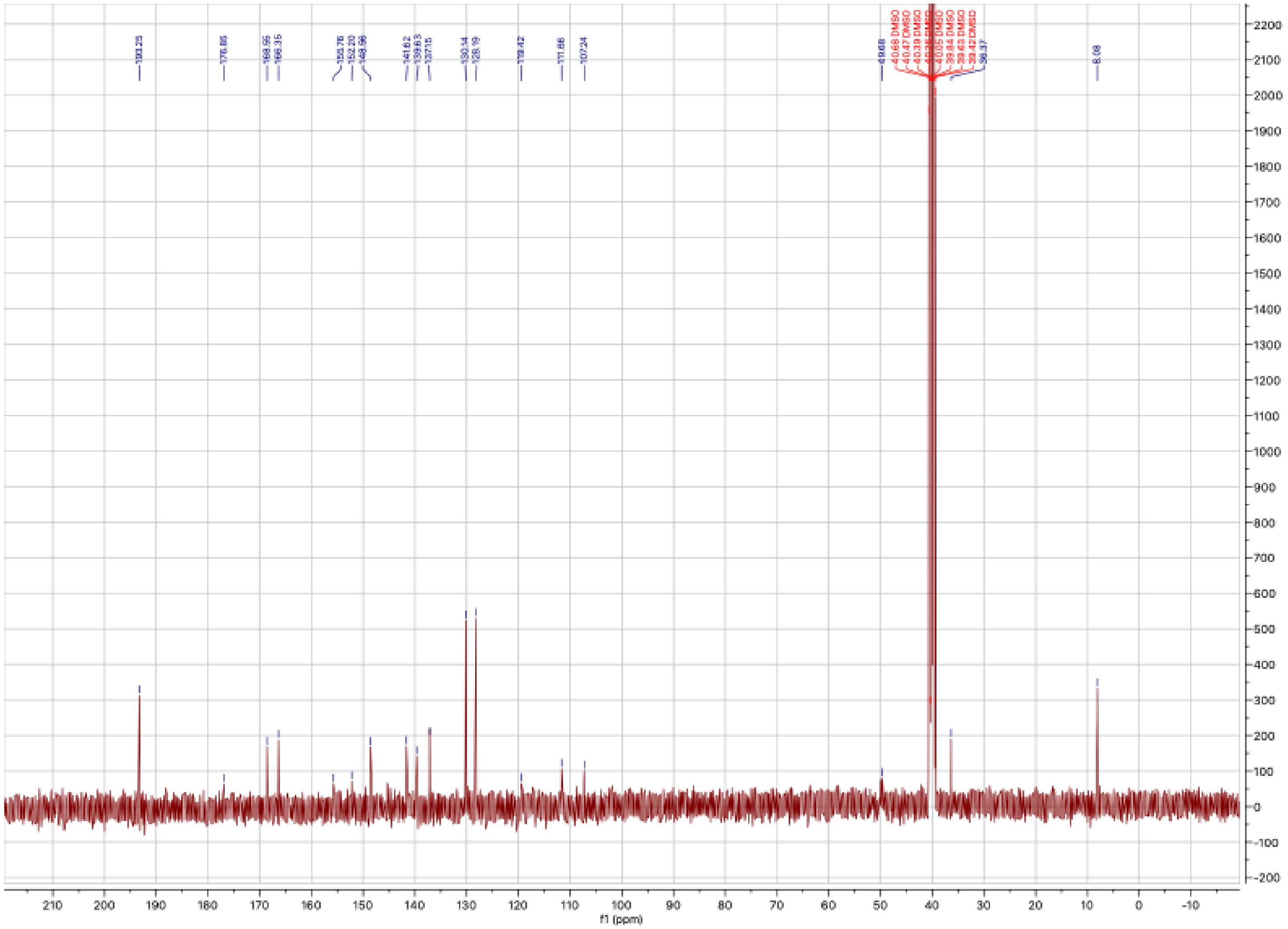

(E)-7-(4-(4-((2-((2-(2-naphthamido)-5-bromobenzoyl)-L-tryptophyl)hydrazineylidene)methyl)benzoyl)piperazin-1-yl)-1-cyclopropyl-6-fluoro-4-oxo-1,4-dihydroquinoline-3-carboxylic acid (**4**)

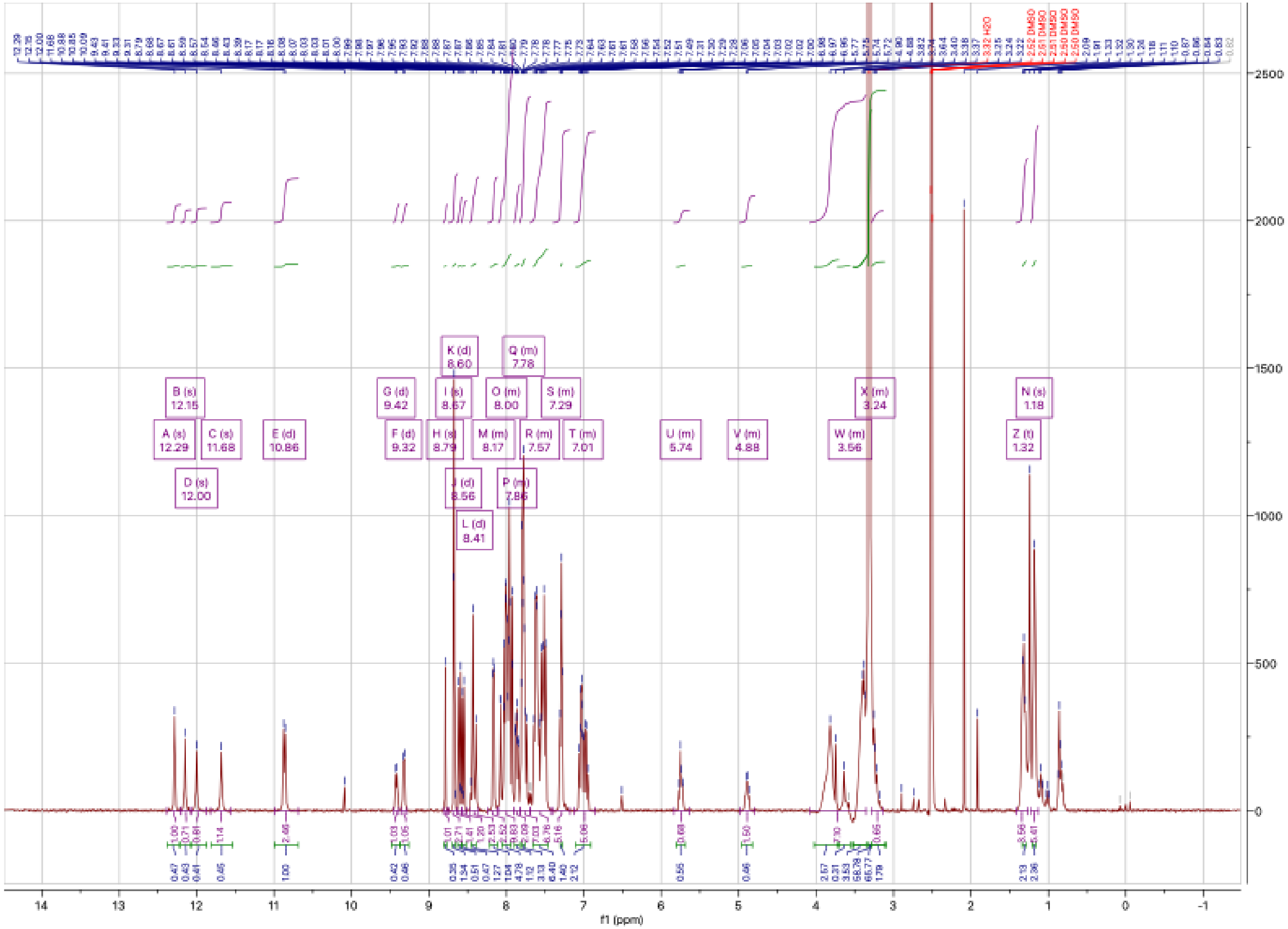

(E)-7-(4-(4-((2-((2-(2-naphthamido)-5-bromobenzoyl)-L-tryptophyl)hydrazineylidene)methyl)benzoyl)piperazin-1-yl)-1-cyclopropyl-6-fluoro-4-oxo-1,4-dihydroquinoline-3-carboxylic acid (**4**)

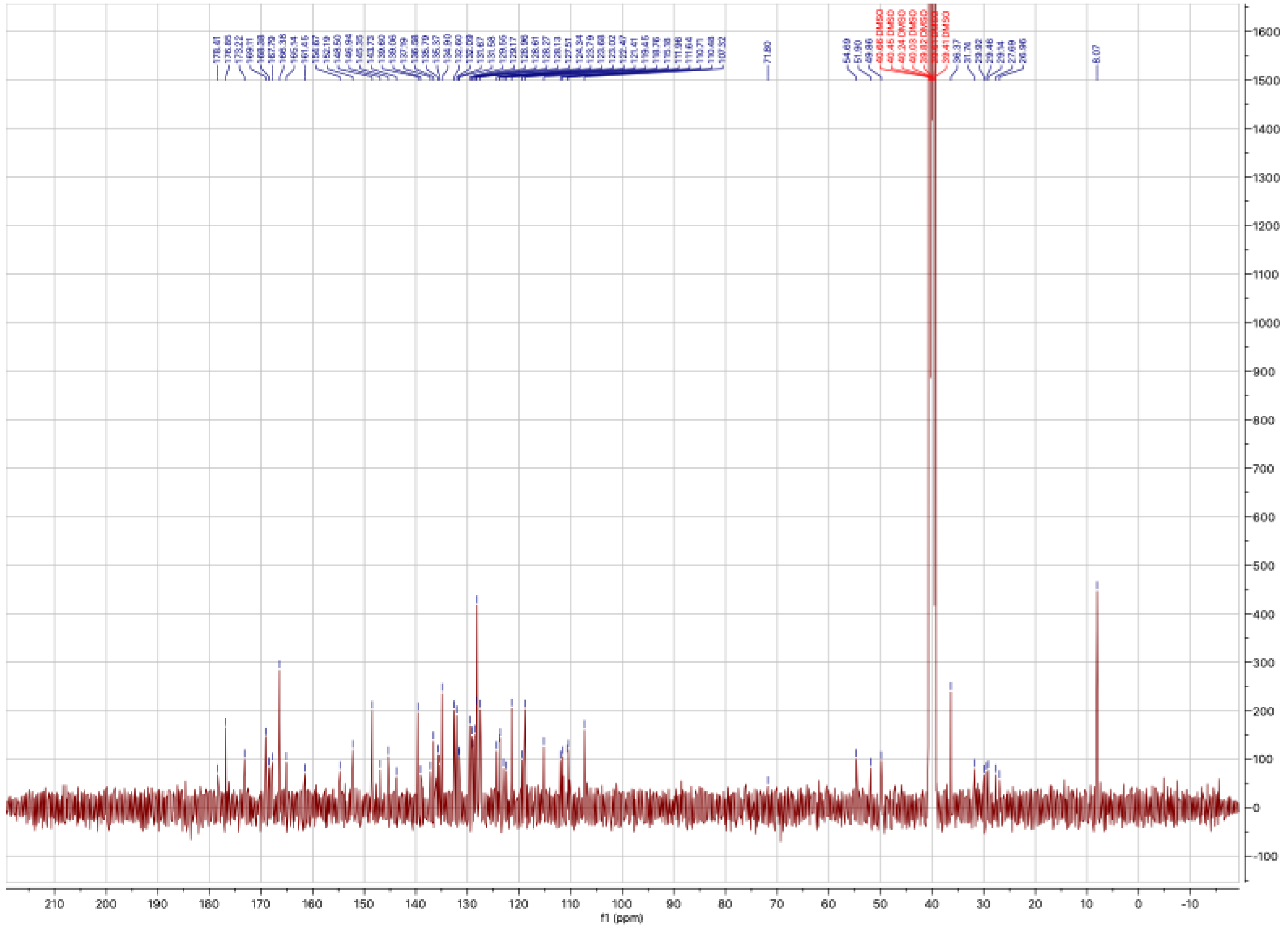

4-methoxybenzyl 3-((4-((2-(2-naphthamido)-5-bromobenzoyl)-L-tryptophyl)piperazin-1-yl)methyl)-8-oxo-7-(2-phenylacetamido)-5-thia-1-azabicyclo[4.2.0]oct-2-ene-2-carboxylate (**18**)

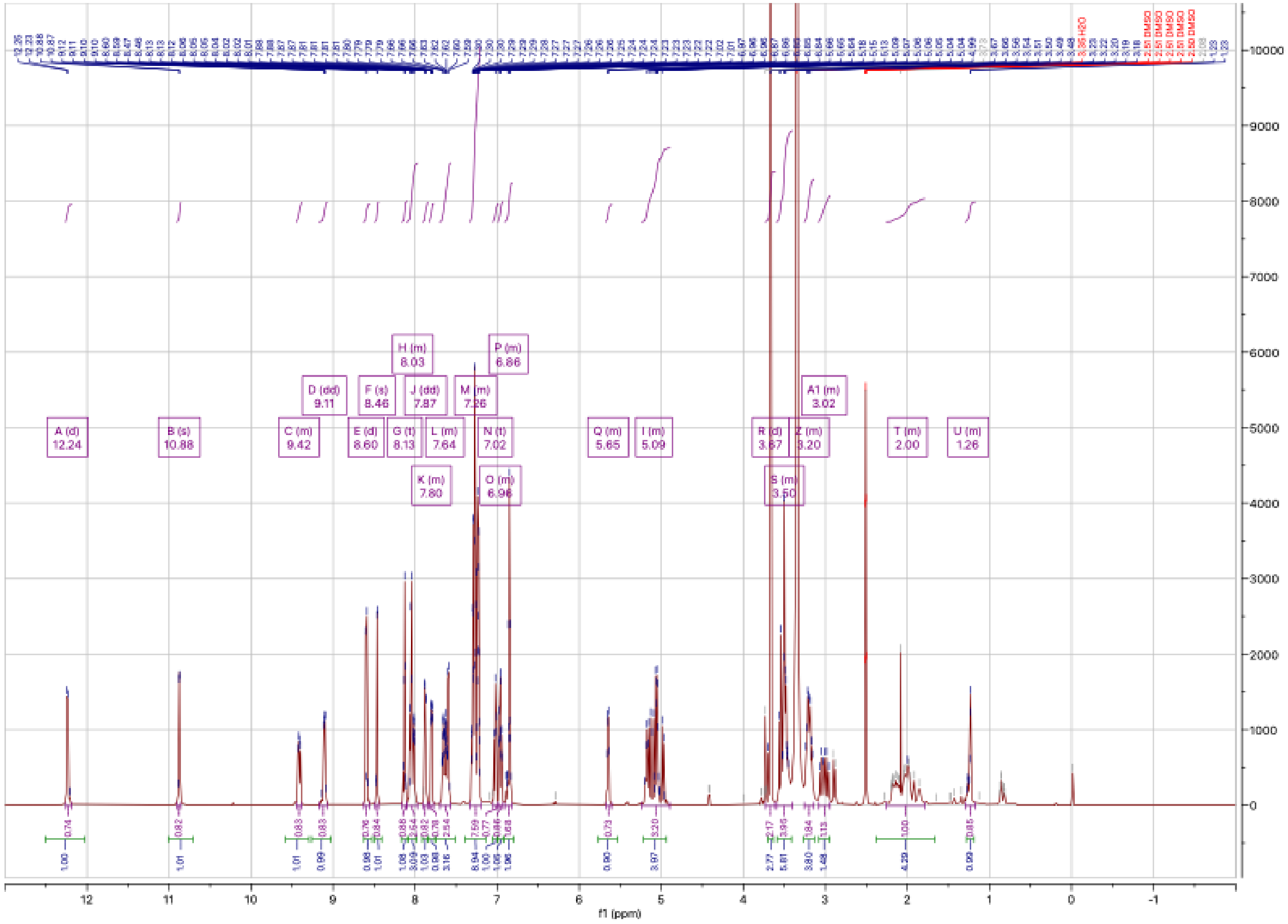

4-methoxybenzyl 3-((4-((2-(2-naphthamido)-5-bromobenzoyl)-L-tryptophyl)piperazin-1-yl)methyl)-8-oxo-7-(2-phenylacetamido)-5-thia-1-azabicyclo[4.2.0]oct-2-ene-2-carboxylate (**18**)

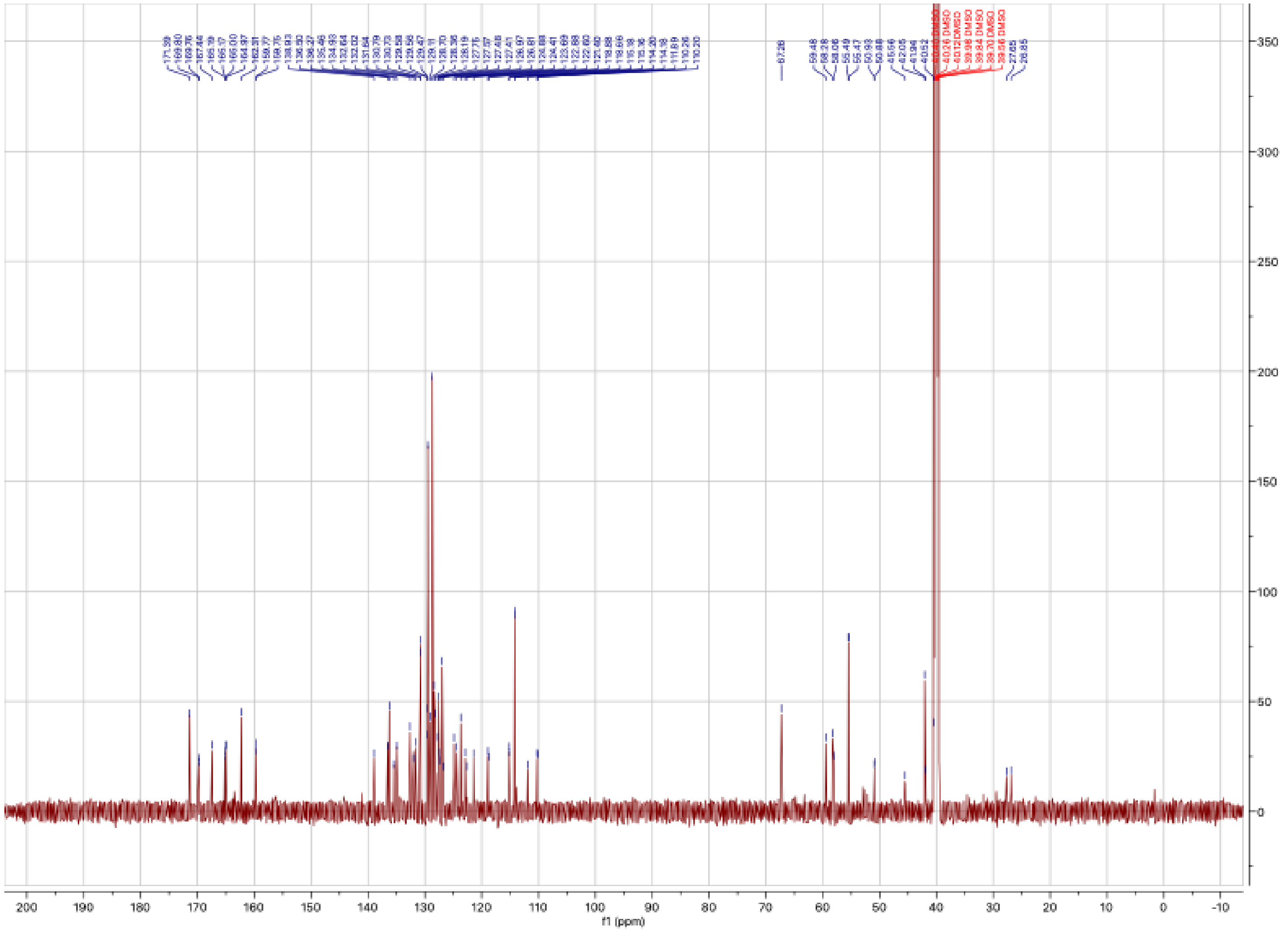

3-((4-((2-(2-naphthamido)-5-bromobenzoyl)-L-tryptophyl)piperazin-1-yl)methyl)-8-oxo-7-(2-phenylacetamido)-5-thia-1-azabicyclo[4.2.0]oct-2-ene-2-carboxylic acid (**5**)

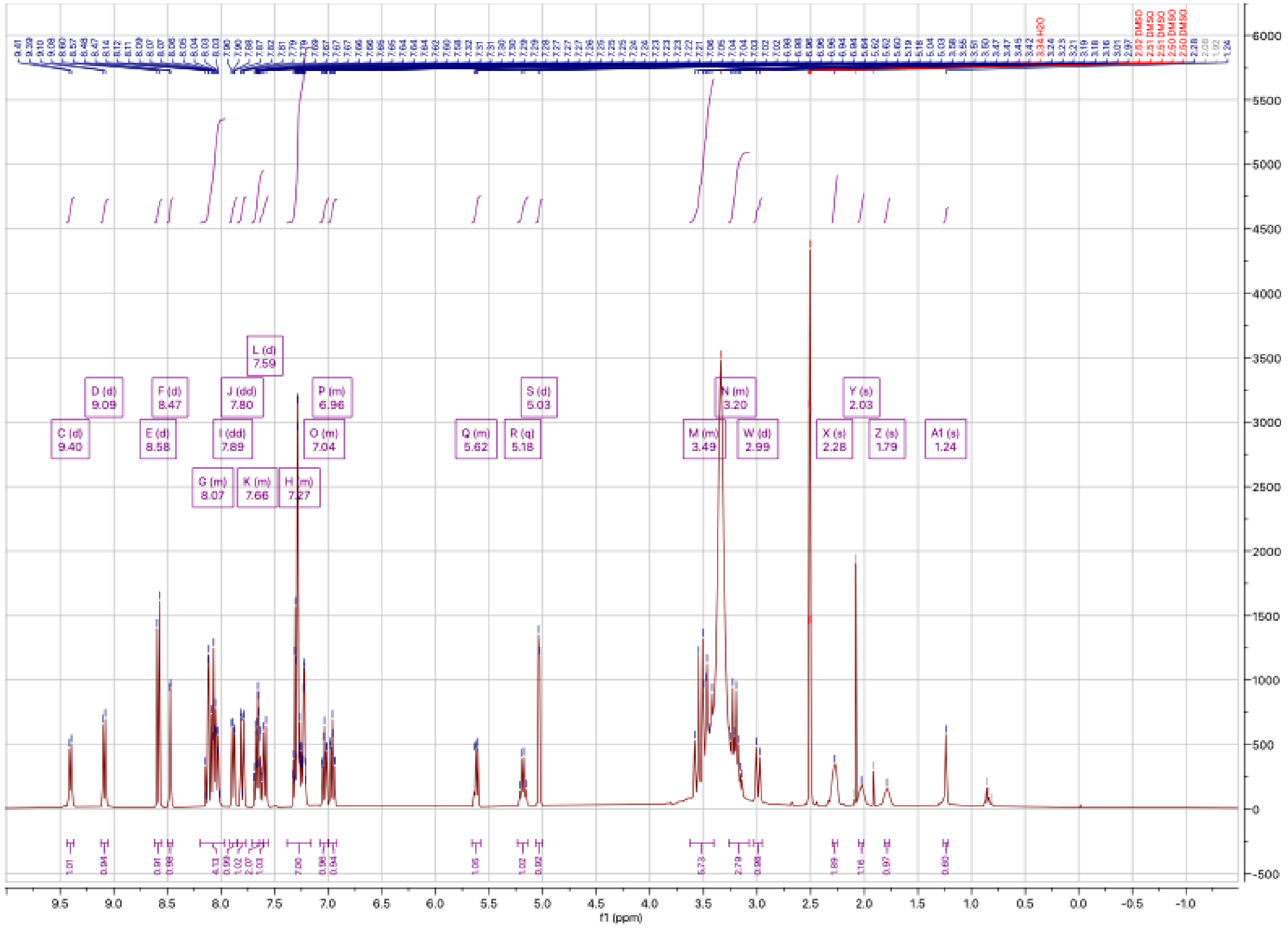

3-((4-((2-(2-naphthamido)-5-bromobenzoyl)-L-tryptophyl)piperazin-1-yl)methyl)-8-oxo-7-(2-phenylacetamido)-5-thia-1-azabicyclo[4.2.0]oct-2-ene-2-carboxylic acid (**5**)

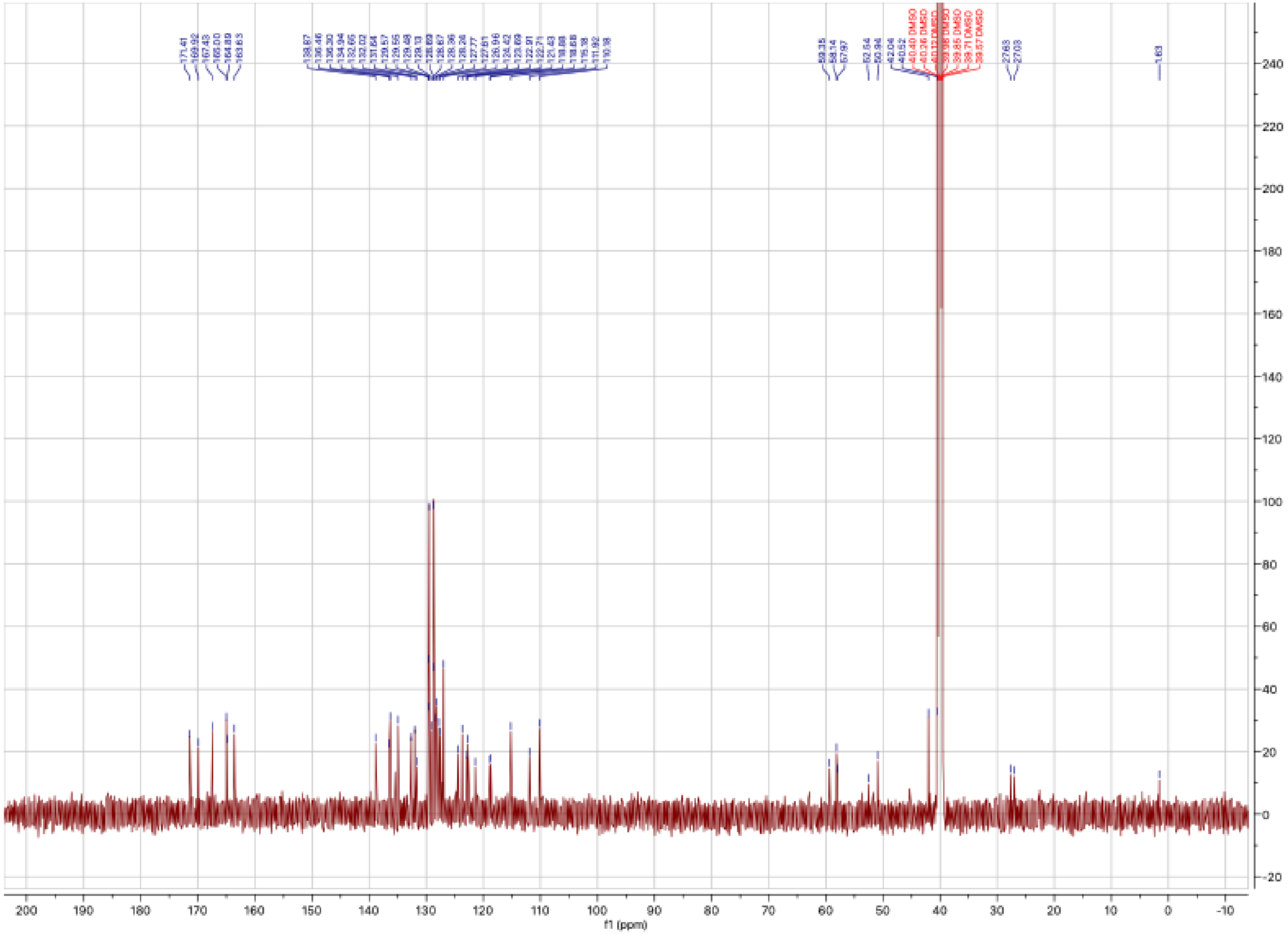

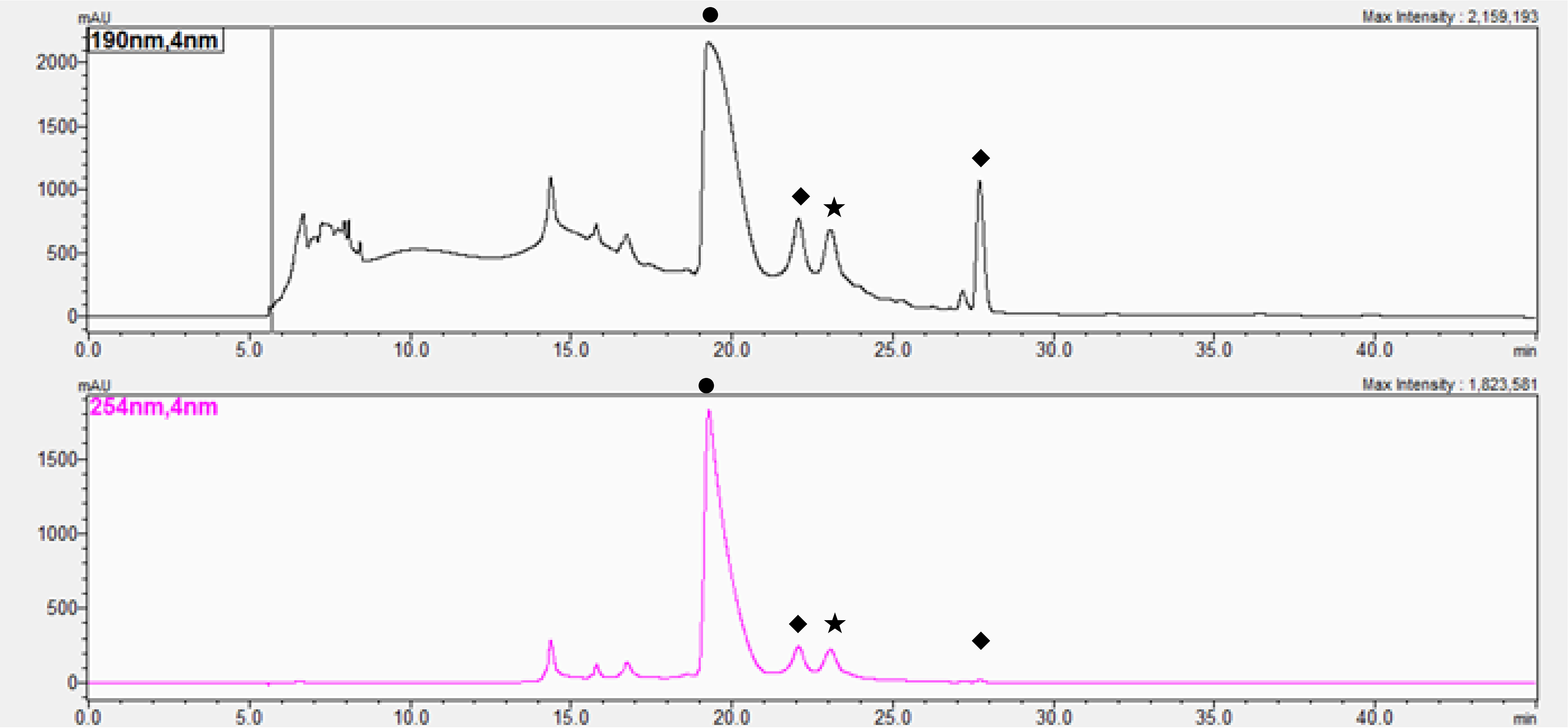

*Figure S1: LCMS trace of conjugate **5** after exposure to pellicinase enzyme for 5 hours: ● = free amine cleaved product **1b**; ★ = conjugate **5**; ◆ = degradation product.*
